# Multi-Functional Self-Adhesive Porous Patches with Anisotropic Charges for Abdominal Wall Repair

**DOI:** 10.34133/research.0945

**Published:** 2025-11-05

**Authors:** Shangrui Rao, Wenzhao Li, Hongzheng Li, Jianhua Lu, Minyu Zhou, Danna Liang, Letian Meng, Yongdong Yi, Bingzi Zhu, Puxiang Lai, Yin Jin, Ji Lin, Yu Wang, Weijian Sun

**Affiliations:** ^1^ Department of Gastrointestinal Surgery, The Second Affiliated Hospital of Wenzhou Medical University, Wenzhou 325027, China.; ^2^ Department of Biomedical Engineering, The Hong Kong Polytechnic University, Hong Kong SAR 999077, China.; ^3^ Department of Gastrointestinal Surgery, The First Affiliated Hospital of Wenzhou Medical University, Wenzhou 325027, China.; ^4^ Wenzhou Institute, University of Chinese Academy of Sciences, Wenzhou 325001, China.

## Abstract

Biomedical patches have garnered extensive value in abdominal wall repair. Major challenge still remains in the improvement of structure, function, and their active ingredients for realizing long-lasting and effective therapeutic outcomes. Herein, we develop a self-adhesive porous patch with anisotropic charges via a simple integrally molding method. The bottom side of the patch with the dense structure exhibits anti-adhesion behavior that acts as a protective barrier for surrounding organs and tissues. Comparatively, owing to the loose porous structure with gradient arrangement, the top side of the patch enables the removal of interfacial water on the tissue surface, facilitating to achieve unique wet self-adhesive properties. Notably, benefitting from the enrichment of negatively charged carboxyl (-COOH) functional groups on the top side of the patch, positively charged pro-inflammatory cytokines can be adsorbed and neutralized on the wound surface, thereby improving the adverse microenvironment. In vivo animal experiments have demonstrated that compared to the commercial patches, the designed patch has the ability to prevent postoperative adhesion and infection, greatly improving abdominal wall repair. These results offer substantial guidance for promoting the clinical translation of abdominal wall defect treatment.

## Introduction

Abdominal wall defects, commonly caused by congenital developmental abnormalities, trauma, surgical complications, etc., are a globally recognized challenge that leads to an enormous burden on the economy [1–4]. The complexity lies in severe tissue adhesions and restricted regeneration, attributed to uncontrolled inflammation with associated cytokines [5,6]. However, there remains a significant gap of appropriate patches in clinical practice. Currently, widely used polypropylene (PP) and polydioxanone-coated (PCO) meshes are simple, hydrophobic polymer materials that lack functional partitioning, offering neither anti-adhesion nor pro-regenerative properties. Furthermore, their absence of inherent self-adhesion means that they rely on sutures, which extends the duration of procedures, compromises interfacial stability, and increases the risk of postoperative displacement. Additionally, their limited ability to modulate the local inflammatory and healing microenvironment hampers long-term repair and tissue integration. As an advanced solution, biomedical patches encapsulated with biological factors have been proposed, achieving sustained drug release for the repair of defects in the abdominal wall [7–10]. Although with great achievements, existing patches with singular structures and functions fall short of achieving optimal therapeutic outcomes [11,12]. In addition, these loaded factors inheriting the nature of chemically unstable pose a huge challenge in preserving their activity, resulting in reduced efficacy [13–16]. Moreover, most of the currently developed patches lack adhesion function, relying on surgical suture or additional tapes, which contains time-consuming processes and needs professional requirements [17–19]. Therefore, it is of immense significance to create novel multi-functional biomedical patches for addressing abdominal wall defects.

**Fig. 1.**
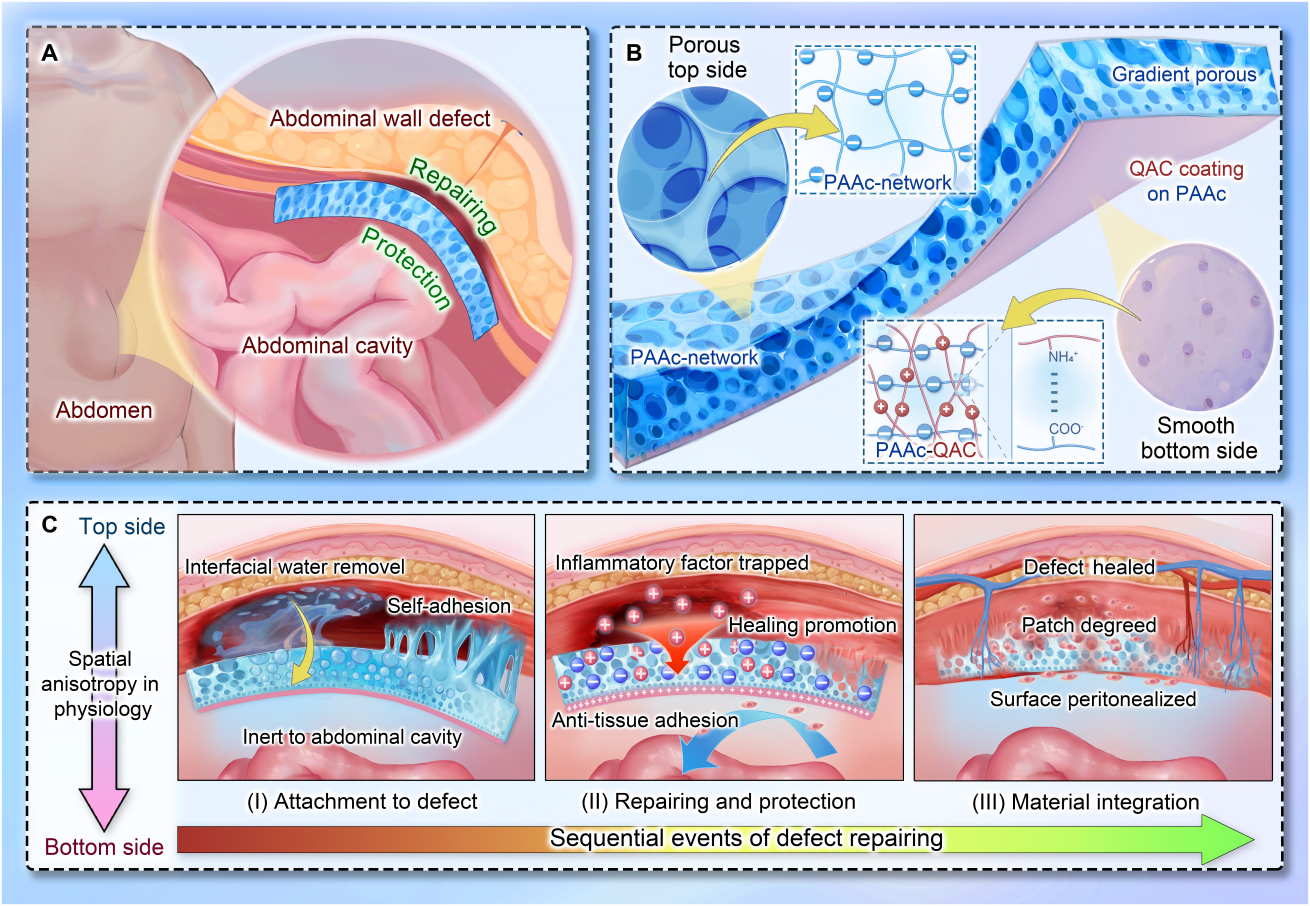
Multi-functional patch for abdominal wall defect repair. (A) Abdominal wall defects present challenges in repair and tissue adhesion on each side. Patches are applied to the defect in situ for repairing and protection. (B) Structure and composition of the patch. Its main matrix is a PAAc network. The top side has large pores, while the bottom side is relatively smooth with a QAC coating. Internally, patches have gradient pores. (C) Spatiotemporal anisotropic roles of the patch: (I) The top side adsorbs interfacial water, achieving self-adhesion; the bottom side is inert without adhesive force. (II) The porous and negative charges on the top side traps positively charged inflammatory factors, promoting healing. The bottom side prevents cell and tissue adhesion. (III) New tissue grows into the pores of the top side, while the bottom surface undergoes peritonealization. The patch eventually partially degrades.

In this paper, we present multi-functional self-adhesive porous (MFP) patches with anisotropic charges for abdominal wall repair, as shown in Fig. 1. Self-adhesive hydrogels, as an attractive alternative to traditional sutures, have a critical role in cases of wound closure, hemostasis, and tissue engineering [20–28]. Notably, it has been reported that the adhesion performance can be greatly improved through a strategy of introducing porous structures with specific surface area [29–31]. However, most reported systems remain focused on isolated properties and are not tailored to the complex clinical conditions of abdominal wall repair. Additionally, to be well-known, the pro-inflammatory cytokines are positively charged, and their excessive release can exacerbate inflammation [32–36]. Charge manipulation has been a promising way for reducing their expression, which establishes a “charge trap” through materials with negative charges and further forms electrostatic adsorption, thereby improving the inflammatory environment [37]. Moreover, the surface charge properties of materials not only influence the adsorption behavior of inflammatory cytokines but also impact macrophage polarization (M1/M2 phenotypes). This dual mechanism regulates immune-related pathways such as Toll-like receptor (TLR)/nuclear factor κB (NF-κB), exerting profound effects on the local immune microenvironment [38–40]. Thus, it is highly expected that the integration of adhesive hydrogels and charge manipulation will contribute to designing a novel promising patch system.

Herein, we used an integral molding method to fabricate an anisotropic porous patch with the desired features for the treatment of abdominal wall defects. Such patches adopted acrylic acid (AAc) as the basic monomer and dimethyl silicone oil as the emulsifier. Taking advantage of ultraviolet (UV) light polymerization, the resultant patches with asymmetric porous structures and anisotropic charges were obtained. Specifically, the bottom side of the patches coated with quaternary ammonium chitosan (QAC) had a dense structure acting as a physical barrier to prevent the development of adhesions. In contrast, the top side of the patches exhibited the loose porous structure with gradient arrangement, providing unique wet self-adhesion properties, which further aided in eliminating interfacial water from the tissue surface. More interestingly, negatively charged carboxyl (-COOH) functional groups were abundant on the top side of the patches, which could adsorb and neutralize positively charged pro-inflammatory cytokines on the wound surface, thereby modulating the local microenvironment. Based on these characteristics, we demonstrated the practical application of patches for abdominal wall defect healing and adhesion prevention in vivo. These results indicated that the proposed multi-functional patches would have important value in the biomedical field.

## Results and Discussion

**Fig. 2.**
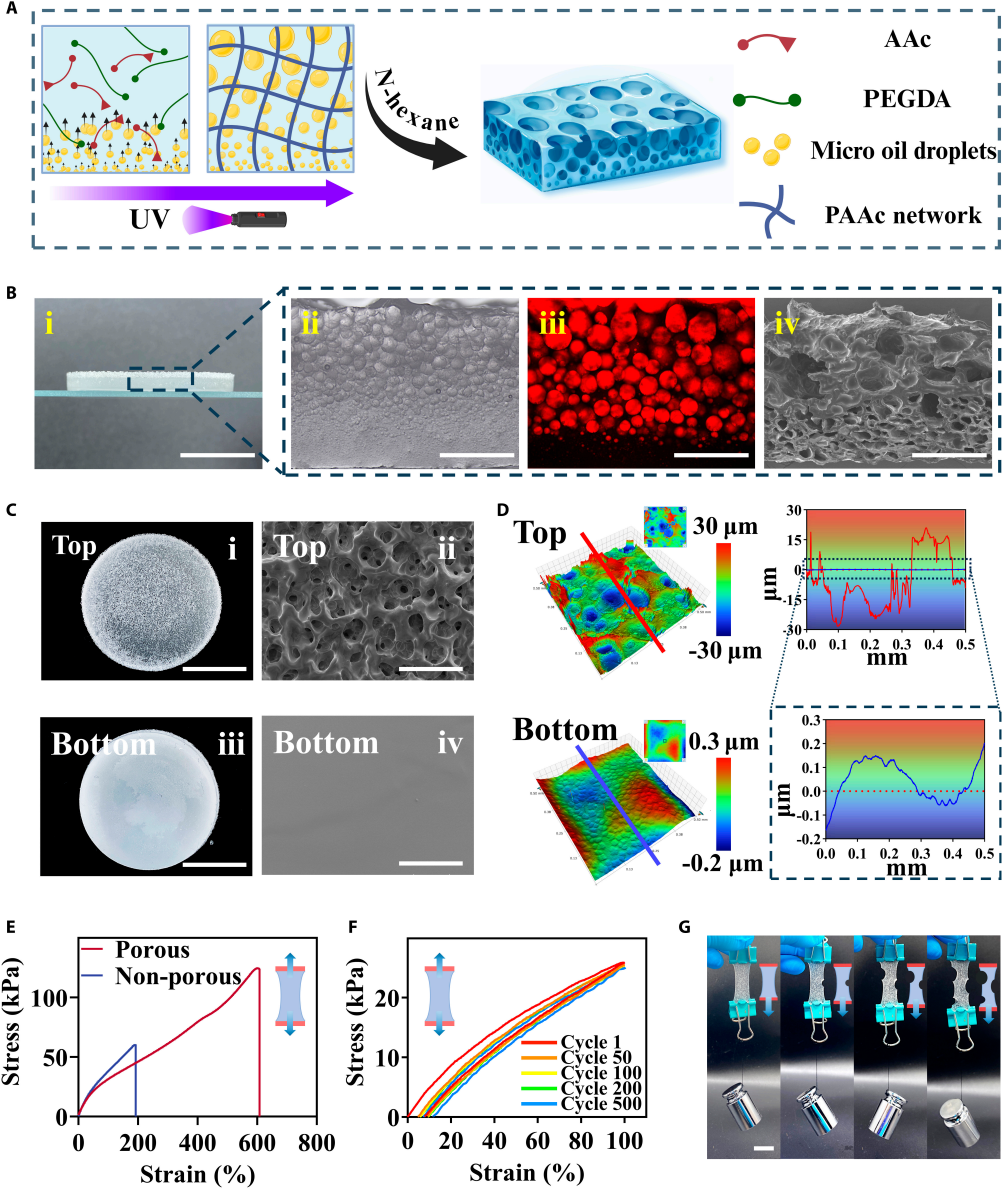
(A) Schematic of MFP preparation. (B) (i) Vertical cross-section image of the porous patch. (ii) Gradient pores observed under an optical microscope. (iii) Gradient pores observed under a confocal microscope. (iv) Gradient pores observed under SEM. (C) Photographs and SEM images of the top and bottom sides. (D) 3D optical profilometer of the top and bottom sides. The red and blue lines indicate the cross-section for height distribution analysis. (E) Stress–strain curve of the patch with/without porous structures. (F) Tensile cyclic stress–strain curve of porous patch. (G) Images of mechanical test of the porous patch. Mechanical testing to evaluate hydrogel crack resistance. Scale bars, 500 μm (B, ii to iv, and C, ii and iv), 1 cm (Bi and C, i and iii), and 2 cm (G).

In this experiment, the MFP patches were fabricated through the integral molding method. Specifically, a metastable emulsion was constructed consisting of the aqueous and oil phases. The aqueous phase was a hydrogel precursor, with AAc as the monomer, along with an appropriate crosslinker and initiator. The oil phase contained dimethyl silicone oil and an emulsifier. Under suitable oscillatory emulsification conditions, the metastable emulsion containing numerous oil droplets was formed. The oil droplets within the emulsion underwent 2 simultaneous processes: forming larger droplets via coalescence upon collision, and rising driven by buoyancy. According to Stokes’ law, larger oil droplets ascend more rapidly, ultimately resulting in gradient rearrangement of oil droplets from large to small [41]. By photo-crosslinking the hydrogel network, a hybrid patch was formed, which preserved the dynamic gradient oil droplet structure. After removing oil droplets, the resultant patch with gradient porosity was formed in situ (Fig. 2A and Fig. S1).

To optimize this dynamic process for the desired structure, the volume ratio of the oil phase (emulsifier:dimethyl silicone oil) was adjusted and the emulsion conditions were monitored over time (Fig. S2). The results showed that fewer emulsifier [emulsifier:oil = 1:10 (v/v)] led to rapid coalescence (Fig. S2A), while more emulsifier [emulsifier:oil = 1:2 (v/v)] resulted in stable flocculation (Fig. S2C). When the volume ratio of emulsifier and dimethyl silicone oil was 1:6, the metastable emulsion could be formed (Fig. S2B). The microstructures of the patches obtained at 3 concentrations of oil phase were detected. It was found that gradient oil droplets only existed at the volume ratio of emulsifier and dimethyl silicone oil of 1:6 (Fig. S3ii), while excessively large pores (Fig. S3i) and nonheterogeneous pores (Fig. S3iii) were observed in the other 2 cases. Given these, an optimized oil phase volume ratio of 1:6 was used for the fabrication of the patch.

To clearly observe the gradient arrangement structure of oil droplets, a thicker patch embedded with oil droplets was fabricated and its cross-section was characterized Fig. 2B. Optical microscopy (Fig. 2Bii) and fluorescence imaging (Fig. 2Biii) revealed a gradient change of oil droplets from a few micrometers at the bottom to several hundred micrometers at the top. After removing the oil droplets, the porous structure with the gradient arrangement was easily identified from the cross-sectional image (Fig. 2Biv). Such gradient arrangement structure would have significant influence on the surface of the patch. To investigate this, scanning electron microscopy (SEM) was employed to examine the morphology of both surfaces, showing distinct characteristics on the top and bottom (Fig. 2C). A rough porous appearance was observed on the top side of the patches, whereas the bottom side exhibited a relatively smooth and dense morphology (Fig. S4). The pores on the top side were larger than 100 μm, whereas pores on the bottom side were smaller than 10 μm. The anisotropic microstructure was confirmed by 3-dimensional (3D) optical profilometry, where different surface heights were assigned distinct color mappings (Fig. 2D). Quantitatively, line-scan analysis along the red marker on the top surface revealed a significant height variation of approximately 60 μm, whereas the height variation along the blue marker on the bottom surface was minimal, around 0.5 μm. It is noteworthy that both profiles were obtained using the same method; however, to enhance visibility, pseudo-color mappings with different vertical ranges were applied, further highlighting the contrast in surface topographies. These results proved that the acquired patch had anisotropic sides and gradient arrangement porous structures.

Additionally, the mechanical properties of patch were systematically optimized, which is critical for abdominal wall repair [12,42]. As an implant, patch needs to accommodate the flexibility of soft tissue while also resisting adverse mechanical disturbances throughout the healing process [43,44]. First, the effect of the amount of the crosslinker poly (ethylene glycol) diacrylate (PEGDA) on the mechanical properties was explored. It was observed that better breaking strain was gradually achieved with the decrease of the PEGDA concentrations. As the amount of crosslinker increased, the breaking strain decreased while the breaking strength increased. Considering the optimal balance between breaking strain (192%) and strength (59.8 kPa) with a modulus of 0.2881 kPa, we determined that the suitable concentration of PEGDA was 0.5 wt % (Fig. S5). Additionally, we confirmed that the porous structure significantly improved the mechanical performance of the patch. Specifically, as shown in Fig. 2E and Fig. S6, compared with homogeneous hydrogels, MFP exhibited higher fracture strain (>600%) and toughness, highlighting the significant role of the pore architecture in stress distribution and energy dissipation, consistent with previous reports [45,46]. A successive cyclic tensile test was conducted to assess its durability and recoverability. The results showed that under a stretching strain of 100%, the patch could endure 500 stress-relaxation cycles with only a slight reduction in tensile stress (Fig. 2F). We also observed that this porous hydrogel exhibited strong crack resistance (Fig. 2G), which may be attributed to its gradient-oriented structure that helps distribute stress more evenly throughout the material, thereby reducing its sensitivity to cracks. In summary, the porous patch displayed outstanding mechanical properties, which would significantly benefit its application in abdominal wall repair.

**Fig. 3.**
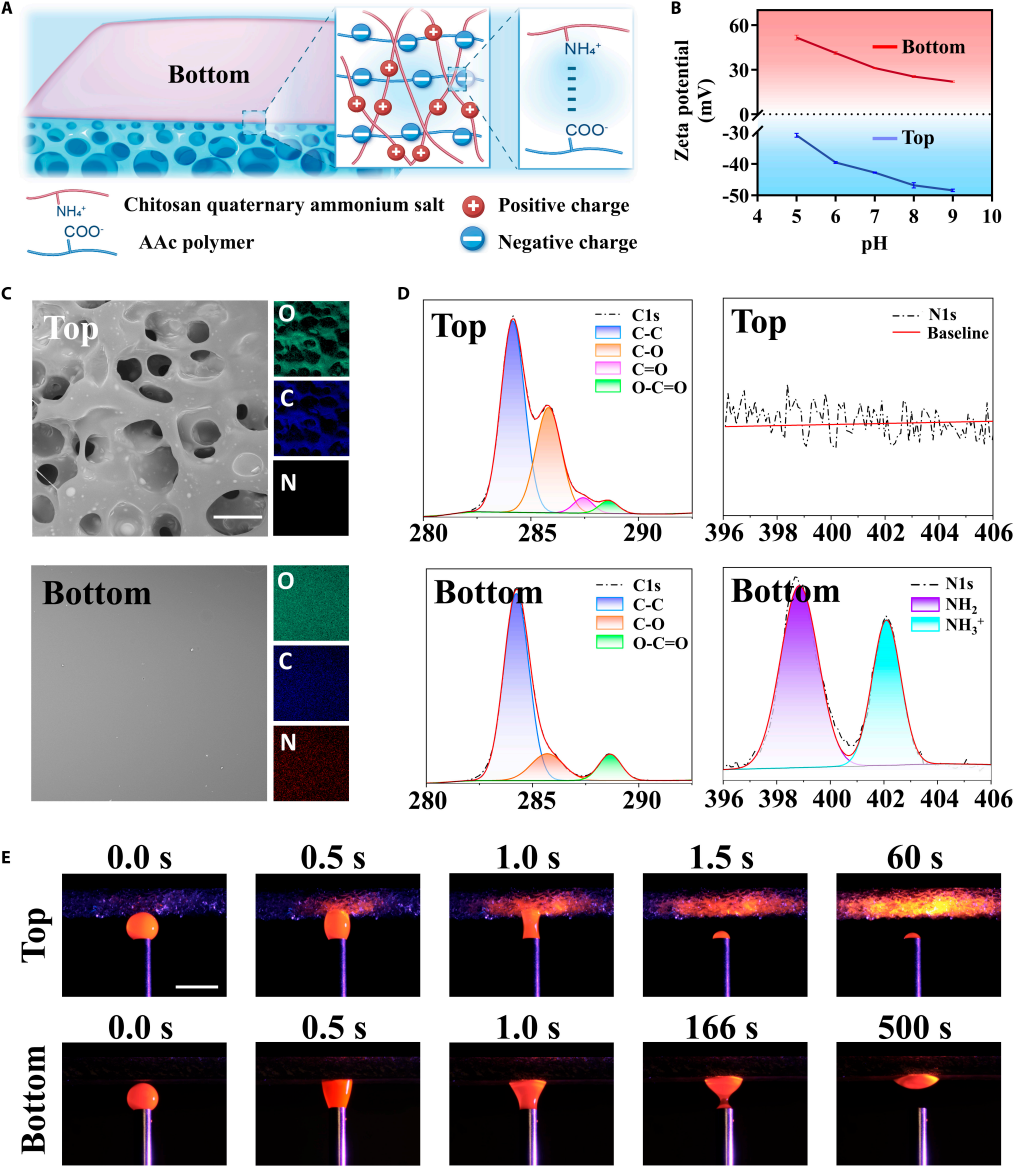
(A) Schematic diagram of chemical structures of MFP. (B) Zeta potentials at the top and bottom sides of MFP. (C) Surface element analysis of both surfaces of MFP. (D) Peak-fitting XPS spectra in the C1s and N1s regions of the 2 surfaces of MFP. (E) Images of both sides of MFP contacting with dyed droplet. Scale bars, 100 μm (C) and 2 mm (E).

Furthermore, different charged polymer coatings were introduced onto the bottom side to establish anisotropic charges, inducing anisotropic behaviors such as adhesive/non-adhesive. Specifically, the main component of the patch, polyacrylic acid (PAAc), is rich in -COOH groups, which impart abundant negative charge. We introduced positively charged QAC onto the bottom side of the patch. An interaction could form between the -NH_3_^+^ groups of QAC and the -COOH groups of PAAc, primarily due to ion–ion interactions and hydrogen bonding (Fig. 3A). As a consequence, the porous patches with anisotropic charges were acquired. The different charges on both sides were embodied via the zeta potential (Fig. 3B). The surface element distribution was analyzed, revealing that the nitrogen element, a characteristic of QAC salt, appeared only on the bottom of the patch, further confirming that the charge difference originated from its successful coating (Fig. 3C). Moreover, the valence bonds of the 2 surfaces were examined using x-ray photoelectron spectroscopy (XPS). Compared to the top surface showing no NH_3_^+^, a strong deconvolution of the N1s peak was detected from the bottom surface, indicating that NH_3_^+^ (402.1 eV) was enriched on the bottom surface of MFP. By the way, the peak of C=O (287.4 eV) appeared only on the top surface, which accounted for the -COOH groups (Fig. 3D). All results illustrated that the QAC salt was successfully coated to the bottom surface of MFP, contributing to the formation of anisotropic charges.

Benefiting from the loose porous structure with gradient arrangement, the top side of MFP was endowed with the ability to remove interfacial water or exudates from the tissue surface. To explore this, we used a self-designed simplified platform to quantitatively apply dyed water droplets to both surfaces and monitored the dynamic process. As shown in Fig. 3E and Movies S1 and S2, upon contact with the top side of MFP, the dyed droplet rapidly and fully spread within 1.5 s, progressing through 3 distinct phases: contacting, diffusion, and wetting. This process is primarily driven by multistage capillary forces generated by MFP. The abundant charges also facilitate its interaction with polar liquids. In contrast, the bottom side of MFP had a negligible absorption even after prolonged contacting for 500 s. By removing interfacial water, the top surface of MFP permits adhesion under wet conditions. MFP also shows a controlled and moderate volumetric swelling ratio, ensuring that structural integrity is preserved without any detachment (Fig. S7).

**Fig. 4.**
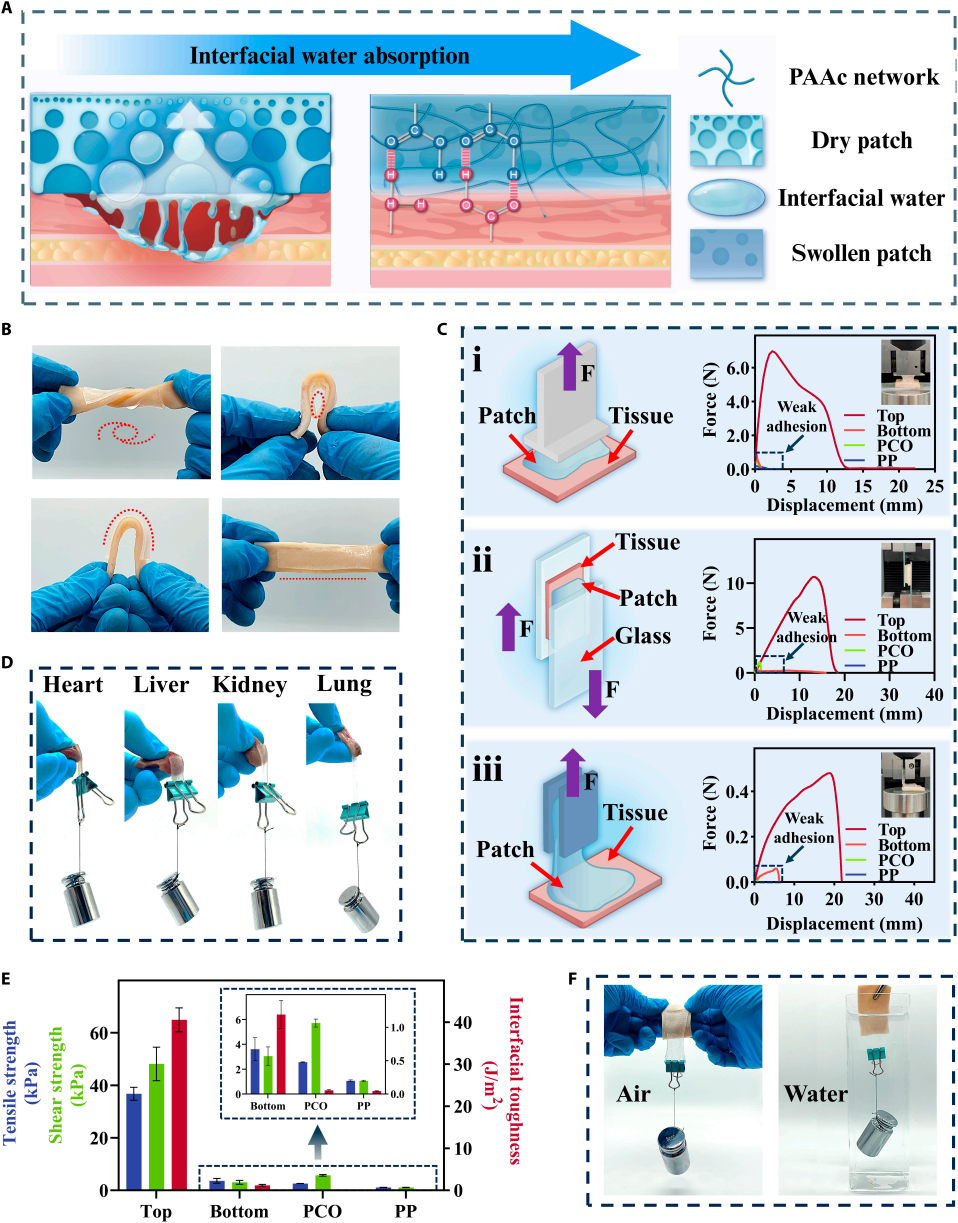
(A) Schematic drawing of the adhesion process on the top face of MFP. (B) Photos of the top side adhered to porcine skin with external forces. (C) Measurement of tensile strength (i), shear strength (ii), and interfacial toughness (iii) of top and bottom side of MFP, PCO, and PP on the porcine skin. The content indicated by the arrow can be found in Fig. S8A. (D) Photos of the top side adhered to various organ tissues. (E) Histogram of the tensile strength, shear strength, and interfacial toughness for each group. (F) Photos of the top side attached to porcine skin in air and underwater.

The MFP’s anisotropic microstructures and charges drive its differentiated adhesive performance. The top side’s superior adhesion stems from its gradient porous structure, with pore sizes decreasing from bottom to top, creating a stable capillary force gradient. This gradient rather than uniform design more effectively guides interfacial water inward for rapid removal, reducing bonding delay and improving initial bonding efficiency [21,41]. The multi-scale pores also increase surface area and interfacial bonding activity, enhancing hydrogen bonds, van der Waals forces, and polymer chain entanglements compared to uniform bulk [47,48]. Specifically, when applied to wet tissues, the top side swiftly removed interfacial water, which is a common cause of other adhesive failures on wet surfaces as it inhibits effective bonding between adhesives and tissues [49–51]. Afterward, the -COOH groups in PAAc form strong molecular interactions, such as hydrogen bonds and Coulombic interactions, with -NH_2_ groups located at the tissue interface. Simultaneously, the hydrated hydrogel establishes robust entanglements at the tissue interface through the diffusion of PAAc polymer chains. Additionally, the high stretchability of the PAAc network and its mechanical dissipation properties further enhance the patch’s adhesion (Fig. 4A). In contrast, the smooth surface of the bottom is unable to remove interfacial water efficiently. Additionally, the interaction between the PAAc network and QAC blocks even crosslinks the free -COOH groups and polymer chains, thus exhibiting anti-adhesive properties.

To evaluate the adhesion performance, MFP was adhered to porcine skin. It was observed that when suffering from a series of deformations including twisting, bending, and stretching, it still robustly adhered to the porcine skin (Fig. 4B). To quantitatively estimate their adhesion properties, we systematically conducted 3 mechanical tests following international standards: tensile tests based on ASTM F2258, lap shear measurements according to ASTM F2255, and peel tests at 180° based on ASTM F2256. Results showed that the top side of MFP established strong adhesion to the tissue, achieving significant strength and toughness across all 3 test modes (Fig. 4C). Specifically, the material achieved tensile and shear strengths of 39.3 and 55.3 kPa, respectively, along with a toughness of 42.3 J/m^2^ in peel mode (Fig. 4E). More intuitively, in tensile mode, it can bear a maximum weight of 1,459 times its own mass (0.5 g), far exceeding the requirements for self-adhesion (Fig. 4Ci). In contrast, the bottom side exhibited significantly lower adhesion performance, with the strength being about 16 times lower and the toughness reduced by approximately 29 times compared to the top side (Fig. 4E and Fig. S8Bii). This adhesion property difference in both sides verified anisotropic adhesive performance of MFP.

Furthermore, commercial patches (PP and PCO) were adopted to further investigate the MFP’s adhesive ability. The adhesion strength of the top side of MFP was approximately 235 and 274 times stronger than PCO and PP, respectively (Fig. 4Ci). In terms of shear and peeling testing, the top side of MFP also showed distinctive adhesion advantages compared to other patches (Fig. 4C, ii and iii, and E and Fig. S8). More broadly, the patch exhibited similarly outstanding adhesion on various organ surfaces, conforming closely and resisting external forces (Fig. 4D). Notably, the top side of MFP not only had extremely strong adhesion in dry environment but also maintained a stable and strong adhesion effect even underwater (Fig. 4F and Movies S3 and S4), providing a solid foundation for its applicability in real-world abdominal applications.

**Fig. 5.**
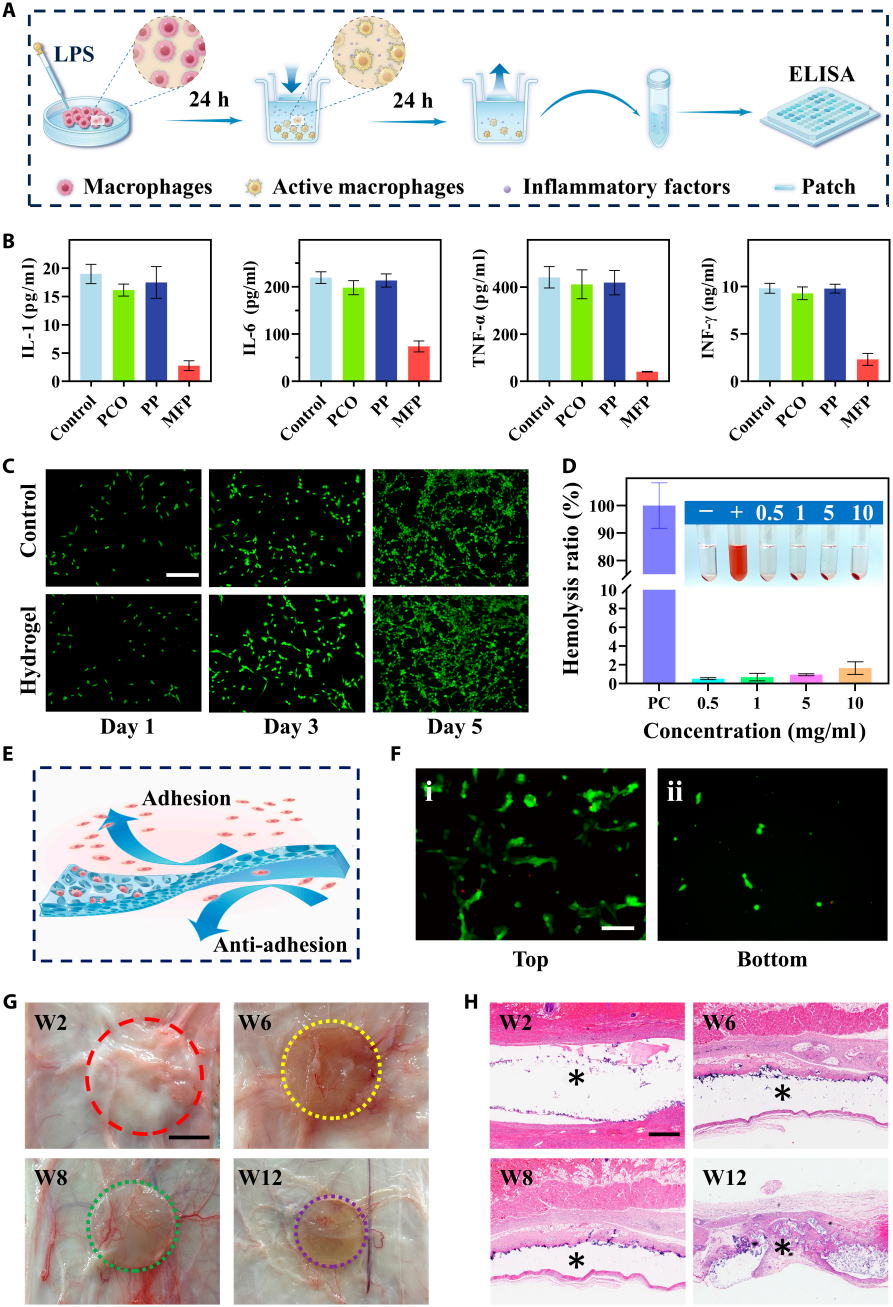
In vitro inflammatory factor adsorption, cytotoxicity, hemolysis test, cell adhesion test, and in vivo biodegradability. (A) Schematic diagram of hydrogel inflammatory factor adsorption test. (B) Concentrations of TNF-α, IFN-γ, IL-6, and IL-1 in the supernatant of cells after 24-h coculture with PCO, PP, and MFP. (C) NIH-3T3 fibroblast live/dead staining images for both the control group and MFP. (D) Hemolysis test of the patch, with inset images illustrating erythrocytes incubated with varying concentrations of MFP. (E) Schematic diagram of the anisotropic cell adhesion behaviors of MFP. (F) Fluorescence images of NIH-3T3 fibroblasts cultured on the top side (i) and bottom side (ii) after 1 d. (G) MFP images were taken every 2 weeks (W) for 3 months after implantation (see Fig. S11B for details). The dashed lines in various colors indicate the boundary of the implanted MFP after absorption at different time intervals. (H) H&E staining images of the subcutaneous patch taken every 2 weeks for 3 months post-implantation (see Fig. S11D for details), with * indicating the implanted MFP. Scale bars, 100 μm (C), 50 μm (F), 5 mm (G), and 1 mm (H).

Abdominal wall soft tissue injuries are typically accompanied by the rapid release of local pro-inflammatory cytokines. M1-polarized macrophages, as the primary source of these cytokines, may sustain persistent postoperative activation, which can lead to excessive inflammatory responses. This prolonged inflammation is considered as a major contributing factor to postoperative visceral adhesions and impaired tissue regeneration [5,40]. Thus, reducing pro-inflammatory cytokines is crucial for controlling inflammation and supporting tissue repair. Many of these cytokines, including interferon-γ (IFN-γ), tumor necrosis factor-α (TNF-α), interleukin-6 (IL-6), and IL-1, carry positive charges. In this study, the top surface of the MFP patch was enriched with carboxyl (-COOH) groups, forming a negatively charged layer that electrostatically adsorbs these cytokines. This “charge trap” effect may modulate macrophage polarization, thereby contributing to an improved local immune microenvironment [52]. To further verify this mechanism, we established an in vitro inflammatory model by stimulating macrophages with lipopolysaccharide (LPS) to induce pro-inflammatory cytokine secretion, followed by co-incubation with patch materials. Enzyme-linked immunosorbent assay (ELISA) was performed to quantify the levels of representative cytokines in the supernatant (Fig. 5A). ELISA results showed that, after 24 h, inflammatory cytokine levels in the MFP group were significantly lower than those in the commercial patch group (Fig. 5B), which is consistent with literature reports that inflammatory cytokines in wound tissue are typically released with high intensity but for a short duration during the early stage, indicating that MFP can effectively achieve the cytokine clearance required for early inflammation control [5,53]. The abundant carboxyl (-COOH) functional groups on the surface of the MFP patch may contribute to this effect by creating a negatively charged microenvironment. Such anionic surfaces have been reported to inhibit M1 macrophage polarization while promoting the M2 phenotype via interference with key signaling pathways such as TLR4/NF-κB, thereby modulating macrophage adhesion, migration, and phenotypic plasticity. These immunoregulatory properties collectively support local immune microenvironment remodeling at the molecular level [54–56]. In light of the above mechanisms, the carboxyl-rich surface and high cytokine-scavenging efficiency of the MFP patch suggest that it not only acts as a physical barrier to inflammation but also may regulate immune cell behavior at the interface to facilitate tissue regeneration.

Given the application of in vivo abdominal wall, the evaluation of biocompatibility of MFP is critical. We cultured mouse embryonic fibroblasts (NIH-3T3) in MFP extract to assess cytocompatibility. As a control, the same cell types were cultured using regular medium. During the 5-d culture period, fluorescence images from live/dead staining indicated that cell morphology appeared normal and consistent in the control and MFP groups (Fig. 5C). Moreover, the Cell Counting Kit-8 (CCK-8) method was used for quantitative statistics. Within 5 d, cell viability increased steadily with no significant difference between groups. Together, these qualitative and quantitative results indicated that MFP had no adverse effect on cells (Fig. S9). Additionally, in vitro hemolysis assays were used to assess hemocompatibility. MFP showed no signs of hemolysis, and the optimal concentration was 10 mg ml^−1^ (Fig. 5D). These findings confirmed that MFP exhibited excellent biocompatibility and was suitable for biomedical applications.

Beyond cytological testing, we further demonstrated the differences in adhesion from a molecular perspective. Because the initial tissue adhesion process is primarily influenced by protein adsorption on the material surface, we examined the protein adsorption behavior of the 2 patch surfaces to elucidate the mechanism of differential tissue adhesion. We therefore examined the protein adsorption behavior of the 2 patch surfaces to clarify the mechanism underlying subsequent cell adhesion and growth. Fluorescein isothiocyanate (FITC)–bovine serum albumin (BSA) assays showed that the porous, hydrophilic, and functionalized top surface of the MFP patch exhibited markedly higher protein adsorption than the dense, quaternary ammonium-coated bottom surface, which demonstrated excellent anti-fouling properties (Fig. S10). This distinct functional asymmetry provides clear structural–functional support for its anti-adhesion applications. Based on these differences in protein adsorption, we next evaluated whether such surface characteristics would lead to variations in cell adhesion and tissue growth. To explore this, in vitro cell-adhesive behavior was evaluated. Considering that fibroblasts are essential components of connective tissue in wound healing, NIH-3T3 fibroblasts were selected to be cultured on both sides of MFP (Fig. 5E). The results indicated that most fibroblasts adhered to and proliferated on the top side of MFP (Fig. 5Fi), whereas only a few were present on the bottom side (Fig. 5Fii). The phenomenon could be ascribed to asymmetric microstructures. Specifically, the top side with large pores (100 μm) allowed fibroblasts to freely enter the pores, providing space and attachment points for cell growth, while the bottom side with few smaller pores (10 μm) prevented fibroblasts from passing through, acting as a protective barrier against adhesion. These results demonstrated the great anisotropic cell-adhesive performance of MFP.

Next, we implanted MFP subcutaneously in rats for up to 12 weeks to evaluate its degradation in vivo (Fig. S11A). Over time, the size and weight of MFP showed a significant decrease (Fig. 5G and Fig. S11B and C). Hematoxylin and eosin (H&E) staining was performed to explore their in vivo degradability, and the implanted MFP showed a gradual reduction within 12 weeks of implantation (Fig. 5H and Fig. S11D). The MFP’s biodegradability in vivo primarily results from the gradual dissolution and breakdown of its crosslinker (PEGDA). It produces hydrophilic oligomers. We also conducted blood tests, including routine blood work and liver and kidney function assessments, to assess any potential systemic toxicity from the degradation byproducts. Over the 12-week study period, blood analysis results of animals implanted with MFP were comparable to those of healthy animals, with no obvious signs of systemic toxicity (Fig. S12).

**Fig. 6.**
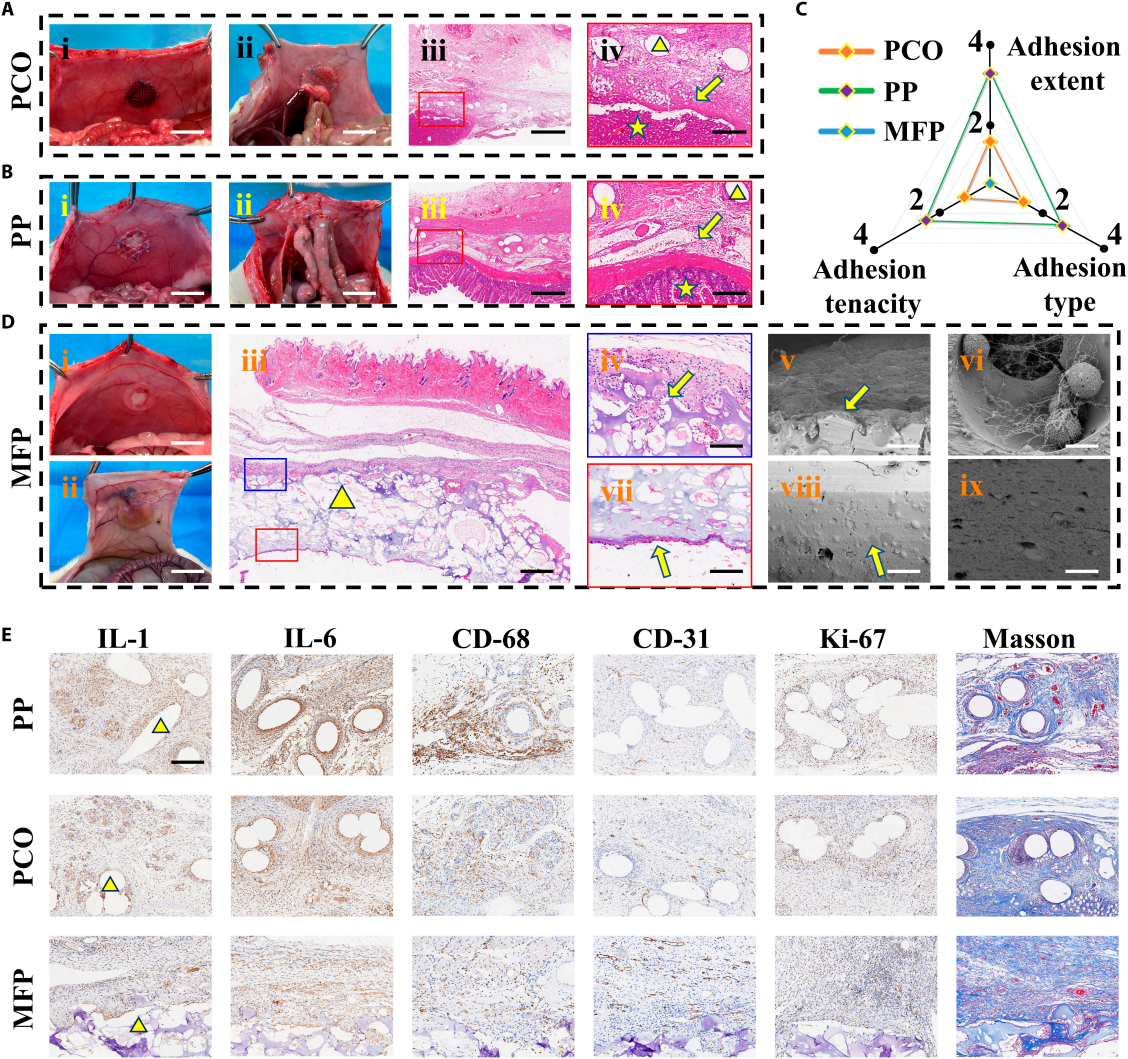
Application of MFP in abdominal wall repair in vivo. (A) Photos (i and ii) and H&E images (iii and iv) of rat abdominal wall repair treated with PCO patches. (B) Photos (i and ii) and H&E images (iii and iv) of rat abdominal wall repair treated with PP patches. The triangle in (Aiv) and (Biv) represents the patches, the 5-pointed star represents the adhered viscera, and the arrows indicate the adhesion boundary. (C) Quantitative assessment of overall visceral adhesion across different treatment groups 2 weeks post-surgery. (D) Photos (i and ii), H&E images (iii, iv, and vi), and corresponding SEM images (v, vi, viii, and ix) of rat abdominal wall repair treated with MFP. The triangle in (iii) represents MFP, (iv) to (vi) are the top side of MFP, and (vii) to (ix) are the bottom side of MFP. (E) Immunohistochemical indices among different treatment groups 2 weeks post-surgery. The triangle represents the patches. Scale bars, 1.0 cm (A, i and ii, B, i and ii, and D, i and ii), 1 mm (Aiii and Biii), 500 μm (Diii), 200 μm (Aiv and Biv), 100 μm (D, iv and v and vii and viii), and 15 μm (D, vi and ix). *n* = 4 for each group.

To explore the practical value of MFP, we conducted abdominal wall defect repair experiments in rats. A rat model of abdominal wall defects was established using a circular punch with a diameter of 1 cm to induce full-thickness muscle-side defects in the abdominal wall. PP and PCO meshes, both available commercially, were chosen as control materials and sutured to the abdominal wall wound. The rats were euthanized 2 weeks after surgery to examine the formation of abdominal visceral adhesions. After opening the abdominal cavity, all abdominal wall defects were successfully repaired. It was found that the commercial patches had varying degrees of adhesion to the intestine, liver, gonadal organs, etc. (Fig. 6Aii and Bii). H&E staining also clearly showed that the commercial patches formed dense adhesions with the liver and small intestine (Fig. 6A, iii and iv, and B, iii and iv). In addition, the foreign body nodules were formed from the commercial patches that resulted from the additional suture fixation, which aggravated abdominal adhesion (Fig. S13). By contrast, no significant adhesions were observed in the MFP group (Fig. 6Dii). More encouragingly, H&E staining and SEM images revealed substantial cell ingrowth on the top surface (Fig. 6D, iv to vi), indicating excellent tissue integration capability. It should be noted that the layered gap observed in Fig. 6Diii is a technical artifact resulting from tissue processing rather than a true adhesion failure. Higher-magnification views (Fig. 6Div) clearly show that the porous structure of the patch remains in close contact with the surrounding tissue. Conversely, dense and smooth bottom side effectively prevented extensive cellular ingrowth into the patch, while a thin side of cells adhered and proliferated, leading to peritonealization of the abdominal surface of the patch (Fig. 6D, vii to ix). These results provided strong evidence that our MFP possessed both effective anti-adhesion and healing-promoting properties.

Additionally, we employed clinical adhesion scoring criteria to quantitatively assess the extent, type, and tenacity of adhesions in each group (Table S1). Upon calculation, the PP mesh had the most severe adhesion, with a score as high as 9 points, and the average score of the PCO mesh was 3.75 points. In sharp contrast, our MFP did not cause visceral adhesion and scored 0 points, showing excellent anti-adhesion effect (Fig. S14). Specifically, when the adhesion extent score was 4.0, the PP mesh scored as high as 3.75 points, which had large-area dense adhesion with the omentum, intestinal tract, and other tissues. In the case of adhesion type score of 3 points, the PP mesh also reached 2.75 points, and its adhered fibrous tissue required sharp peeling to separate the PP mesh. The PCO patch group had a relatively lower degree of adhesion, but it was still not as good as MFP (Fig. 6C). Besides, we also evaluated blood toxicity and histological responses of major organs 2 weeks after surgery in all groups and found no obvious signs of systemic toxicity (Figs. S15 and S16).

We subsequently conducted immunohistochemical assessments of IL-1, IL-6, and cluster of differentiation 68 (CD68), which are pro-inflammatory cytokines (Fig. 6E). According to statistical analysis, the concentrations of CD68, IL-6, and IL-1 expression were considerably decreased in the MFP group (Fig. S17A), indicating MFP’s effectiveness in reducing inflammatory responses. To further elucidate the underlying mechanism, quantitative polymerase chain reaction (qPCR) analysis was performed on abdominal wall tissues at postoperative week 2, focusing on the TLR4/NF-κB inflammatory signaling pathway and immunoregulatory cytokines. The results revealed that TLR4 and MyD88 expression levels in the MFP group were lower than those in the PP and PCO groups, accompanied by decreased IL-6 and increased anti-inflammatory IL-10 expression, suggesting that MFP may exert its effects by suppressing pro-inflammatory signaling and enhancing anti-inflammatory responses (Fig. S18). In view of collagen’s significant role in cell-assisted processes of wound contraction and healing, we explored the effect of the patch on collagen deposition throughout the wound healing process. Masson staining revealed that collagen deposition was most prominent in the MFP group (Fig. S17B). Angiogenesis, considered a key factor in repairing damaged vasculature during wound healing, was assessed by cluster of differentiation 31 (CD31) staining, which indicated increased endothelial cells in the MFP group (Fig. 6E and Fig. S17B). Meanwhile, on postoperative day 14, immunofluorescence staining of abdominal wall tissues revealed clear differences in macrophage polarization among the groups. We selected CD86 and CD206 as markers of M1 and M2 macrophages, respectively. The MFP group displayed pronounced CD206 (green) and relatively weak CD86 (red) signals, indicating a shift toward M2 polarization. In contrast, PP and PCO groups exhibited predominant CD86 expression, reflecting a more pro-inflammatory state (Fig. S19). Quantitative analysis of fluorescence intensity further confirmed these findings. Taken together, these results provide direct evidence that MFP contributes to regulating macrophage polarization, thereby creating a more favorable immune microenvironment for inflammation control and tissue regeneration.

## Conclusion

To conclude, this research effectively developed a multifunctional self-adhesive porous patch with anisotropic charges for abdominal wall repair. MFP was fabricated through a simple integral molding method based on a metastable emulsion, resulting in a PAAc matrix with inner gradient porous structure. It features large pores on the top side and disparate dense surface on the bottom side. Due to the large pores on the top side, MFP could rapidly remove interfacial water from the defect site (within 1.5 s), achieving wet adhesion (over 2,600 times its own weight). Moreover, the PAAc network with -COOH groups could act as a “trap” for positively charged pro-inflammatory cytokines, effectively modulating the local inflammatory microenvironment and potentially influencing macrophage polarization behavior. In contrast, QAC was coated on the bottom side, imparting MFP with anisotropic charges. Together with the dense structure, the bottom side exhibited anti-adhesion properties (^1^/_16_ tensile strength of the top side), forming a protective barrier adjacent to the abdominal cavity. In vivo experiments confirmed the robustness and effectiveness of MFP in adhesion, barrier, tissue repair, and degradation in the full course of abdominal wall defect model, outperforming several commercially available meshes.

Importantly, while many recently reported hydrogels and patches have focused on single aspects such as mechanical strength, adhesion, or immunomodulation, our design uniquely integrates these key functions into a single integrally formed Janus patch, thereby highlighting its advanced nature and direct relevance to the complex therapeutic needs of abdominal wall defect repair. Future studies would be valuable to further investigate the biological mechanisms underlying the material’s effects, such as macrophage polarization and related inflammatory signaling pathways, to deepen understanding and support clinical translation.

## Materials and Methods

### Materials

All chemicals, except where otherwise stated, were procured from Sigma-Aldrich without additional purification. AAc, α-ketoglutaric acid, dimethyl silicone oil, quaternized chitosan, n-hexane, and polyethylene glycol diacrylate (PEGDA) were used in the preparation of the patch. ELISA kits were obtained from Jianglai Bio. Commercial patches were obtained from C. R. Bard Inc.

### Preparation of multi-functional patch

The multi-functional patches were prepared by an integral molding method using AAc as the base. A pre-gel solution was formulated by mixing 30% (w/w) AAc, 0.2% (w/w) α-ketoglutaric acid, and 0.5% (w/w) PEGDA in water, followed by the addition of 100 μl of Tween 80 per ml of this solution, and precursor solutions with varying dimethyl silicone oil concentrations (200 μl, 600 μl, and 1 ml) were prepared. These precursor solutions were vortexed at 3,000 rpm for 1 min and then poured onto a mold equipped with glass spacers for UV curing (395 nm, 12 W) for 5 min. The patch, containing oil droplets, was then soaked in n-hexane and gently cleaned via ultrasonication for 24 h, forming a hydrogel patch with an internal gradient porous structure. Finally, 2 ml of 5 wt % quaternized chitosan solution was spin-coated onto the bottom side of the hydrogel, followed by air drying for 24 h.

### Characterizations

A scanning electron microscope (Hitachi, SU8010) was utilized to investigate the patch. Prior to imaging, the patch was freeze-dried and sputter-coated with platinum. Elemental mapping on the top and bottom surfaces for O, C, and N distribution was conducted using energy-dispersive spectroscopy (Pharos, Phenom). We used an x-ray photoelectron spectroscopy (XPS) instrument (ESCALAB 250XI, Thermo Fisher Scientific) to analyze the surface chemical composition. A 3D optical profiler (Bruker, GT-X) was used to measure the top and bottom surface morphology. The patch cross-section was examined for oil droplet distribution with a high-resolution laser confocal microscope (Nikon, A1). The surface morphology of the top and bottom sides was also captured using an optical microscope (DM1000/DFC295, Leica). The zeta potential of the surface was assessed with Malvern Zetasizer Nano device.

### Mechanical characterization of the patches

Tensile testing was conducted using a universal testing machine (Instron 5944, USA) to assess the mechanical properties of samples. Dog bone-shaped samples were prepared following standard specifications. Then, tensile tests were carried out. During the cyclic tensile tests, samples were stretched to a predefined strain value in each cycle and then returned to their initial length. Each cycle consisted of a stretch and return phase, and the samples were tested for 500 cycles.

### Measurement of adhesive performance

To evaluate adhesive tensile strength, adhesive samples measuring 1.5 cm × 1.5 cm were prepared. The test used a universal testing machine, according to ASTM F2258. For the measurement of shear strength, adhesive samples of the same dimensions (1.5 cm × 1.5 cm) were evaluated according to ASTM F2255. Interfacial toughness was evaluated using 1.5 cm × 3.0 cm adhesive samples with a 1.5 cm × 1.5 cm adhesive area, in accordance with ASTM F2256.

To simulate clinical application on wet tissue surfaces, samples were applied to porcine skin and subjected to a 10 N constant compressive force for 1 min using the testing machine to enhance interfacial interactions, followed by immediate testing. All tests were conducted at a speed of 50 mm/min. Strength was calculated as peak force per unit adhesive area, and toughness was determined as the integral of the force–displacement curve per unit area.

### Absorption behavior of the patch

A custom-built lifting platform combined with a charge-coupled device (CCD) camera was used to monitor the differential water absorption and transfer behavior between the 2 sides of the patch. Specifically, the patch sample was fixed at the top of the device with either the top or bottom side facing down to simulate an abdominal environment. A syringe positioned beneath the sample dispensed 10 μl of dyed phosphate-buffered saline (PBS) (0.1 wt % rhodamine) to simulate interface water droplets. By carefully raising the platform, the syringe was elevated to allow contact between the droplet and the patch surface. The dynamic water transfer process was observed under UV light (*λ* = 254 nm) to stimulate droplet fluorescence.

### Inflammatory factor adsorption test of patch

Macrophages were stimulated in vitro with LPS to induce secretion of inflammatory cytokines and chemokines. The prepared porous patch was placed in a transwell plate with the porous top side facing downward, allowing contact with the cell culture medium for 24 h. The cell culture supernatant was then collected for ELISA to determine cytokine concentrations. Common inflammatory cytokines were measured, using commercial patches as controls.

### In vitro cell compatibility tests

Each well of the 24-well plate was seeded with 2 × 10^4^ NIH-3T3 fibroblast cells. After cells adhered, Dulbecco’s modified Eagle’s medium (DMEM) was exchanged for hydrogel extract, followed by incubation at 37 °C under CO₂ conditions. After 24 h, the cells were rinsed and a diluted CCK-8 solution (1:9 ratio) was added. The samples were incubated for an additional hour in the dark. Cell viability was measured using standard methods.

### Comparative growth of cells on top and bottom sides

All patch samples were cut to fit the size of one well in a 24-well plate. NIH-3T3 cells were cultured on the patch’s both sides, with control groups cultured on polystyrene sides in the same plate wells. The activity of NIH-3T3 cells on both sides was analyzed.

### In vitro hemocompatibility test

Hemolysis tests were performed using rat erythrocytes. The cells were rinsed 3 times with 1× PBS (3,000 rpm for 10 min), and then a 1% (v/v) erythrocyte suspension in PBS was prepared. The hydrogels underwent a 3-h incubation with this suspension at varied concentration levels. Samples were spun for 10 min at a speed of 3,000 rpm, and the absorbance of the resulting supernatant was assessed at OD_540_ (optical density at 540 nm). The rat erythrocytes dispersed in deionized water (1%, v/v) were used as a positive control, and erythrocyte suspension in PBS (1%, v/v) served as the negative control.

### In vivo biodegradability and biocompatibility of the patch

After anesthesia and hair removal, a 2-cm cut was created in the center. The patch was implanted into the subcutaneous space, and the incision was then sutured. Samples of blood were gathered at 2-week intervals over a period of 3 months post-implantation to evaluate toxicity, and the animals were finally euthanized. The patch was photographed, weighed, and histologically examined.

### In vivo abdominal wall defect repair in rat model

After anesthesia and hair removal, a 1-cm full-thickness defect was created in the abdominal wall while preserving the integrity of the skin and subcutaneous tissue. The repair patches were placed between abdominal contents and the abdominal wall. In groups 1 and 2, commercial PP and PCO patches were sutured with 4-0 Vicryl sutures. In group 3, the prepared patch adhered to the defect without sutures. Following the repair, blood samples were taken for examination after 2 weeks. The abdominal wall tissue and surrounding organs were examined for postoperative adhesions and prepared for further analysis.

### Real-time quantitative PCR

Total RNA was extracted from the abdominal wall tissues of the repair region in rats at postoperative day 14 using TRIzol reagent (Invitrogen, USA) according to the manufacturer’s instructions. Reverse transcription of RNA into cDNA was performed using a reverse transcription kit (Takara, Japan). The relative expression levels of IL-6, IL-10, TLR4, and MyD88 were calculated using the 2^−ΔΔCt^ method and normalized to 18*S* expression. The primer sequences (5′→3′) are shown in Table 1.

**Table 1.** Primer sequences used for RT-qPCR

Gene	Forward	Reverse
IL-6	TAGTCCTTCCTACCCCAATTTCC	TTGGTCCTTAGCCACTCCTTC
IL-10	GCTCTTACTGACTGGCATGAG	CGCAGCTCTAGGAGCATGTG
TLR4	ATGGCATGGCTTACACCACC	GAGGCCAATTTTGTCTCCACA
MyD88	TCATGTTCTCCATACCCTTGGT	AAACTGCGAGTGGGGTCAG
18S	TGACGGAAGGGCACCACCAG	GCACCACCACCCACGGAATC

## Ethical Approval

All experiments involving rats were approved by the Animal Care Committee of Wenzhou Institute, University of Chinese Academy of Sciences (approval number: WIUCAS23121401). Male Sprague–Dawley rats (200 to 250 g) were utilized in the experiments (*n* = 4 for each group). Before implantation, patches underwent additional sterilization using UV light for 3 h.

## Data Availability

All data needed to evaluate the conclusions in the paper are present in the paper or the Supplementary Materials. Additional data related to this paper may be requested from the authors.
