# Supplementary figures and images for: Multi-Functional Self-Adhesive Porous Patches with Anisotropic Charges for Abdominal Wall Repair
Source: Research (Wash D C). 2025 Nov 5;8:0945. doi: 10.34133/research.0945 (PMC12586851; doi:10.34133/research.0945)

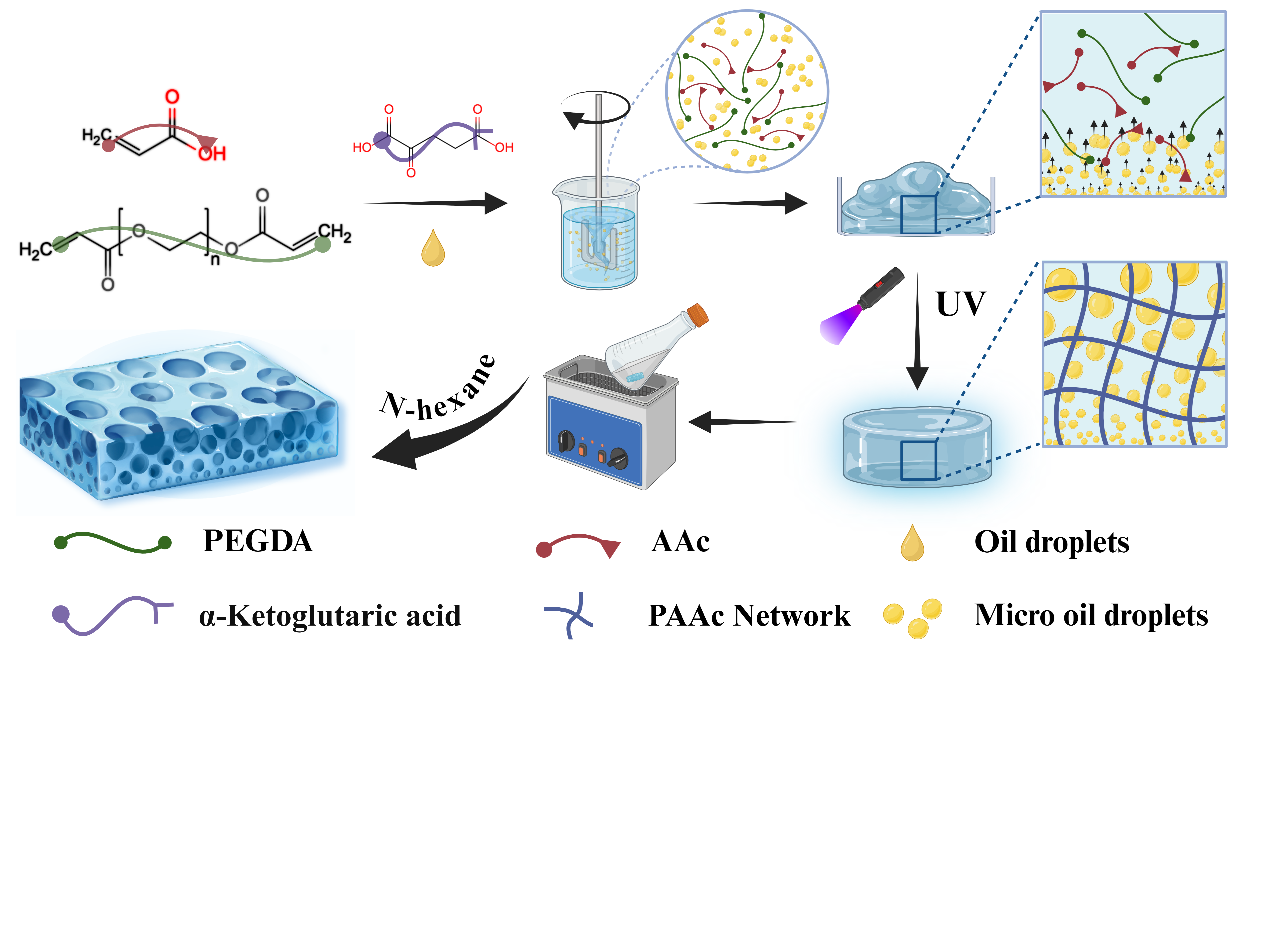

Supplement: Supplementary 1 — Figs. S1 to S19 Table S1 Movies S1 to S4 [file research.0945.f1.zip › Figure S1.TIF]

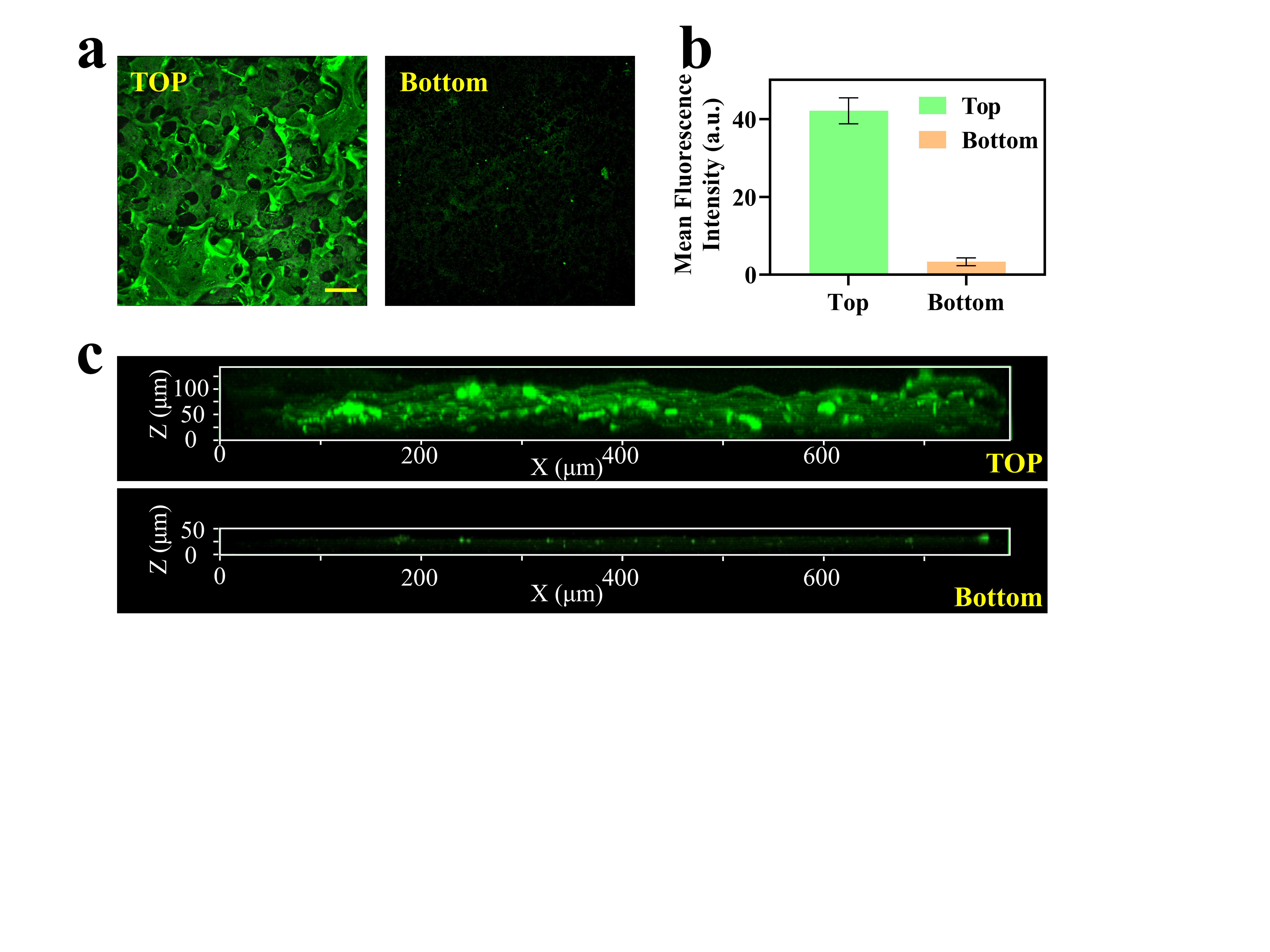

Supplement: Supplementary 1 — Figs. S1 to S19 Table S1 Movies S1 to S4 [file research.0945.f1.zip › Figure S10.TIF]

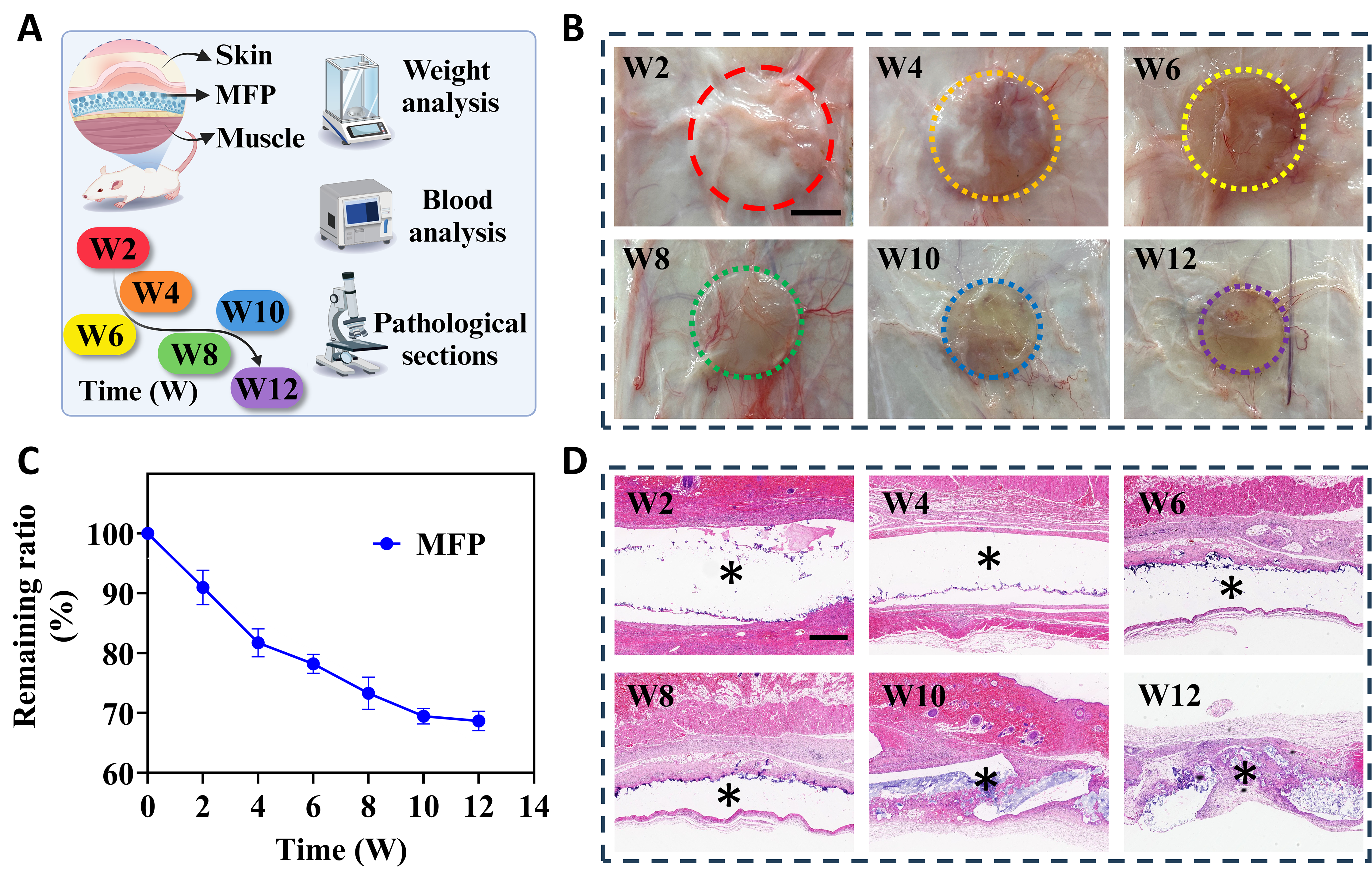

Supplement: Supplementary 1 — Figs. S1 to S19 Table S1 Movies S1 to S4 [file research.0945.f1.zip › Figure S11_highres.png]

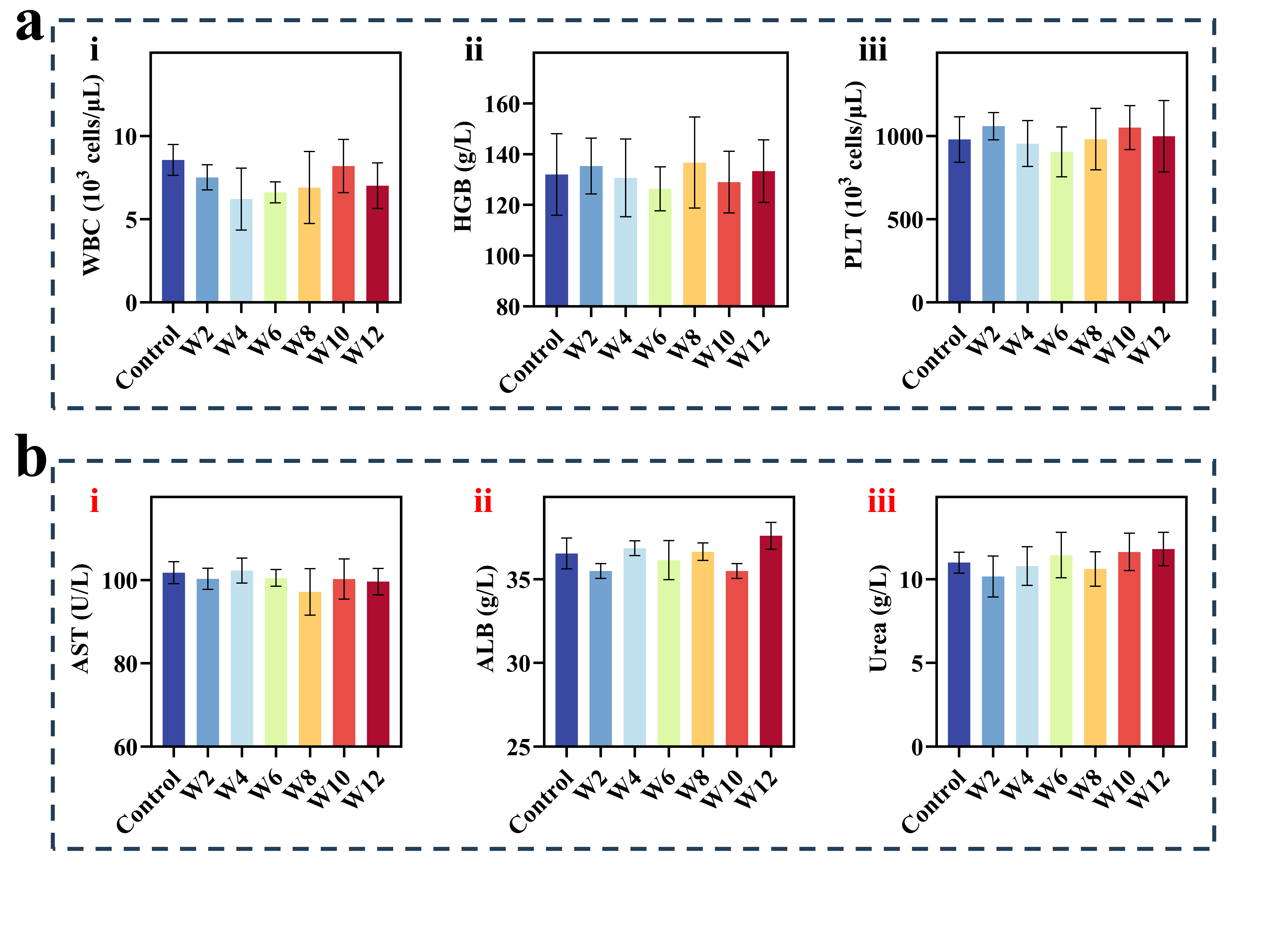

Supplement: Supplementary 1 — Figs. S1 to S19 Table S1 Movies S1 to S4 [file research.0945.f1.zip › Figure S12.TIF]

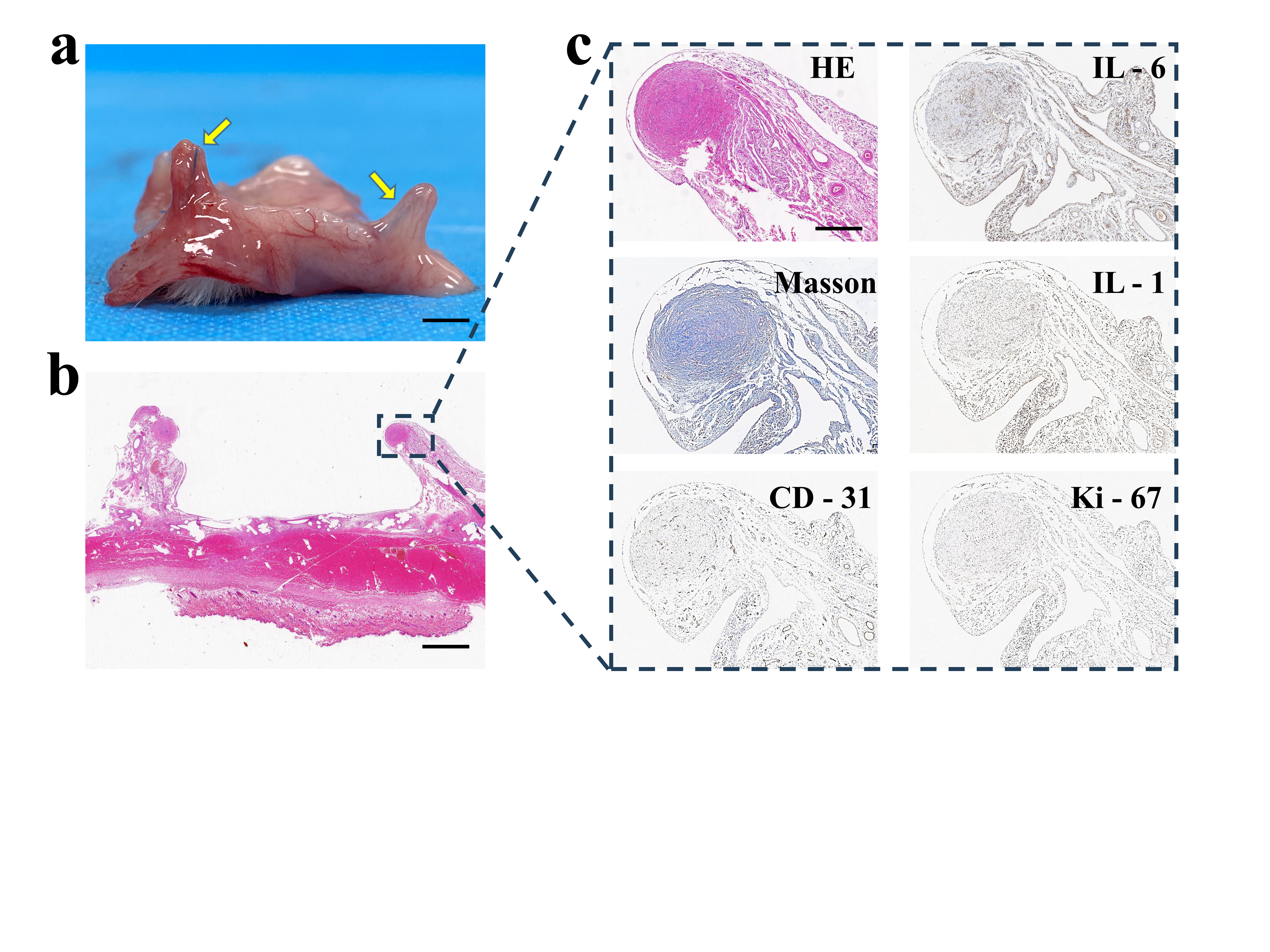

Supplement: Supplementary 1 — Figs. S1 to S19 Table S1 Movies S1 to S4 [file research.0945.f1.zip › Figure S13.TIF]

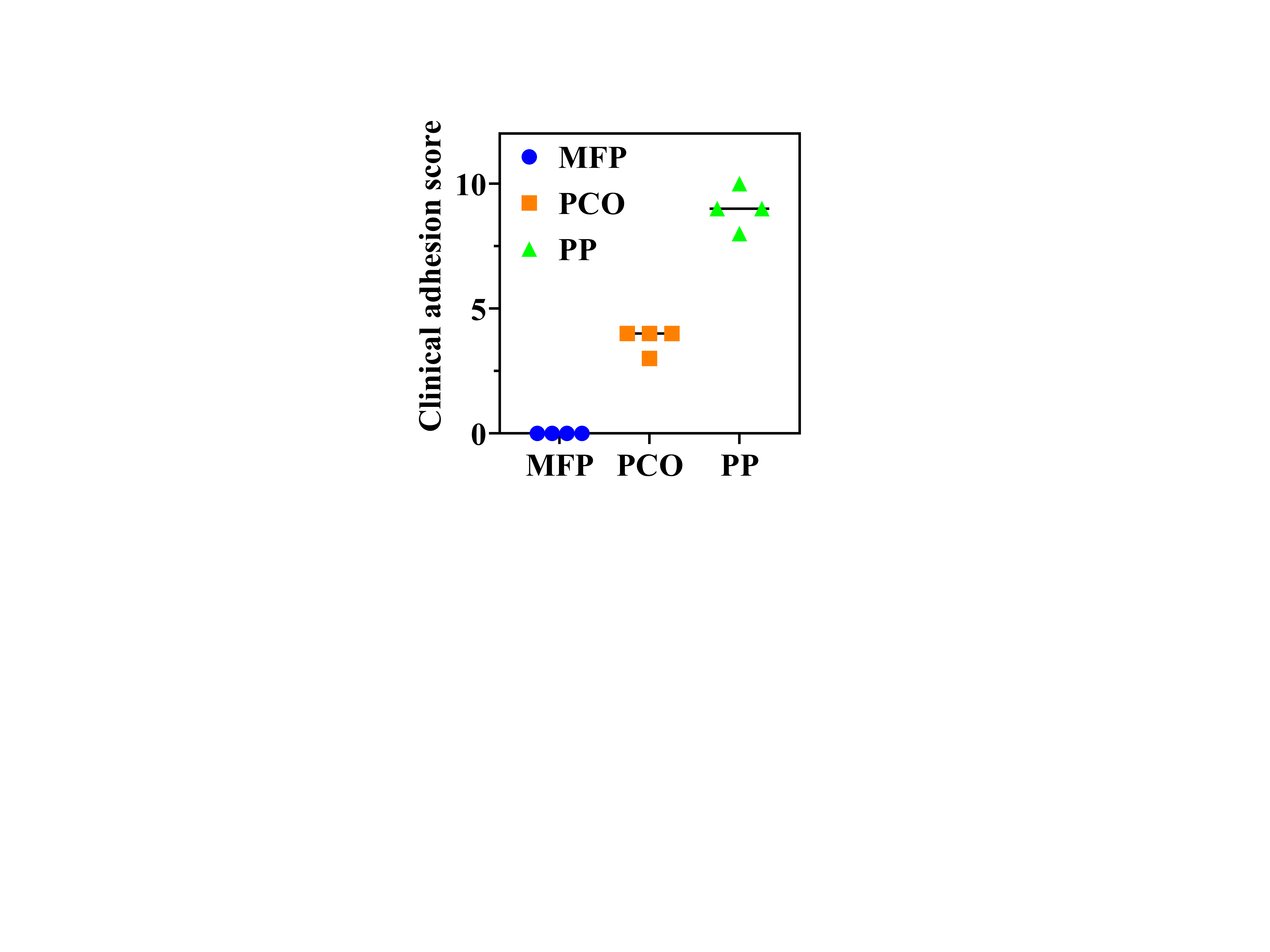

Supplement: Supplementary 1 — Figs. S1 to S19 Table S1 Movies S1 to S4 [file research.0945.f1.zip › Figure S14.TIF]

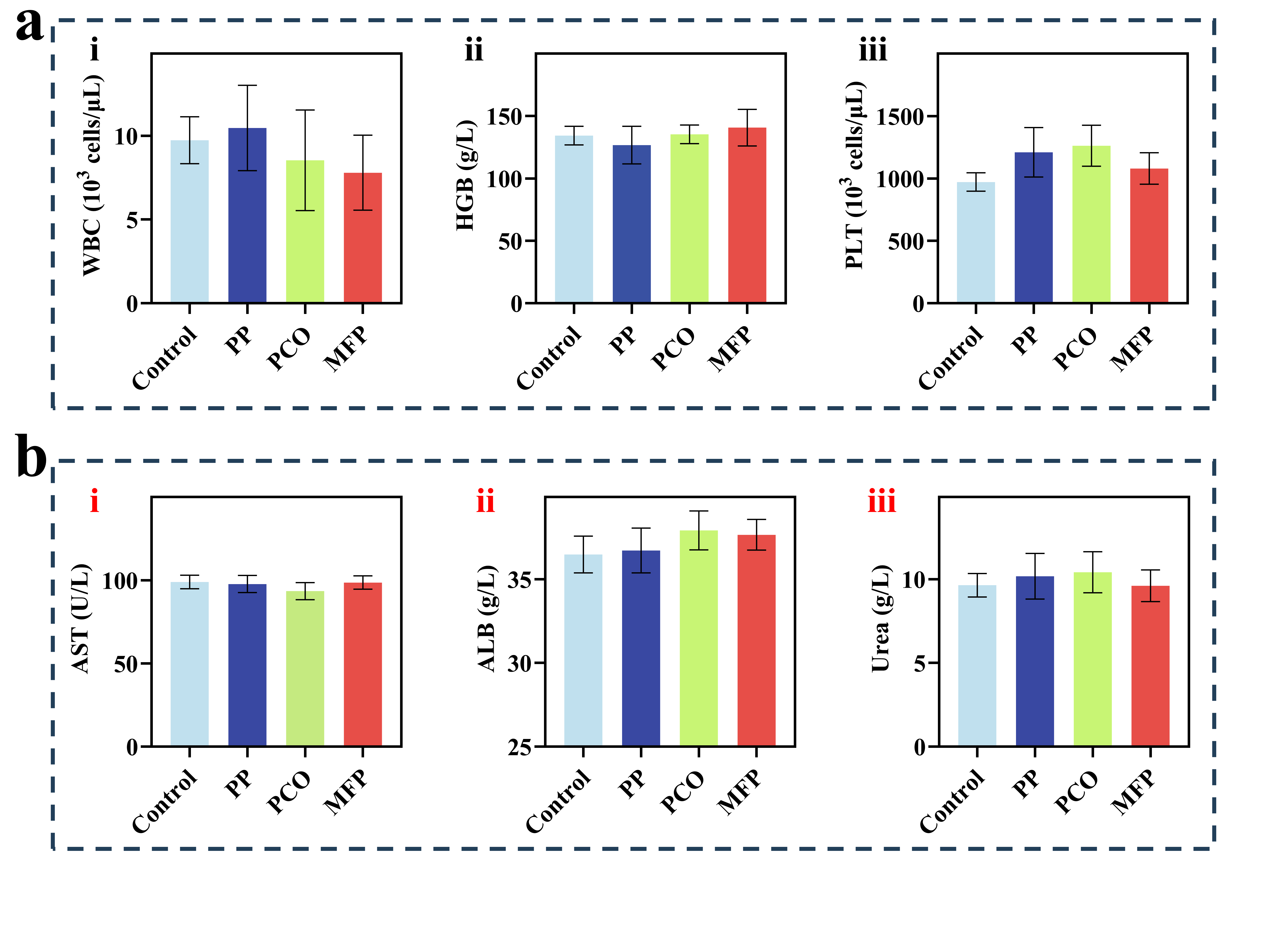

Supplement: Supplementary 1 — Figs. S1 to S19 Table S1 Movies S1 to S4 [file research.0945.f1.zip › Figure S15.TIF]

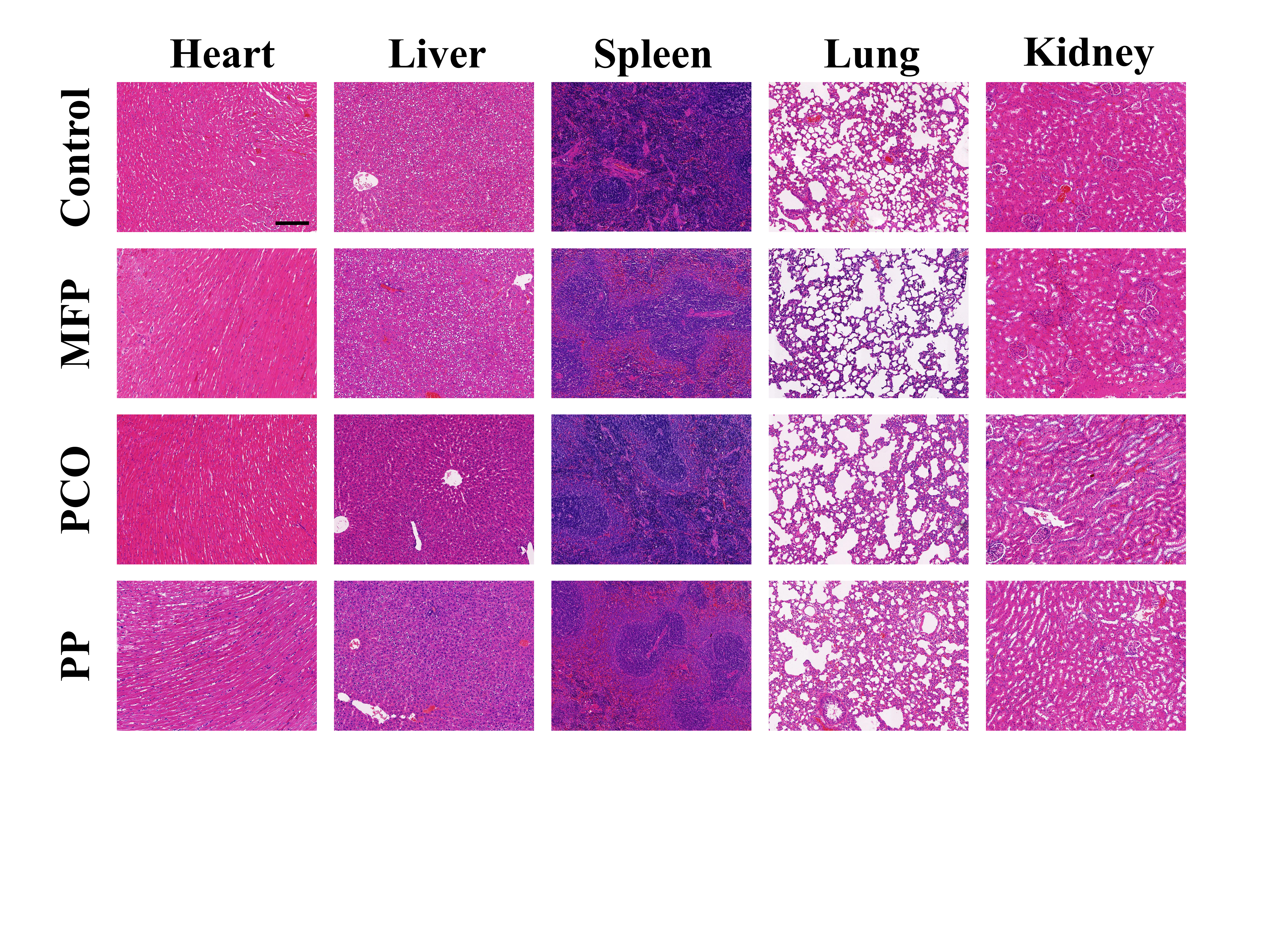

Supplement: Supplementary 1 — Figs. S1 to S19 Table S1 Movies S1 to S4 [file research.0945.f1.zip › Figure S16.TIF]

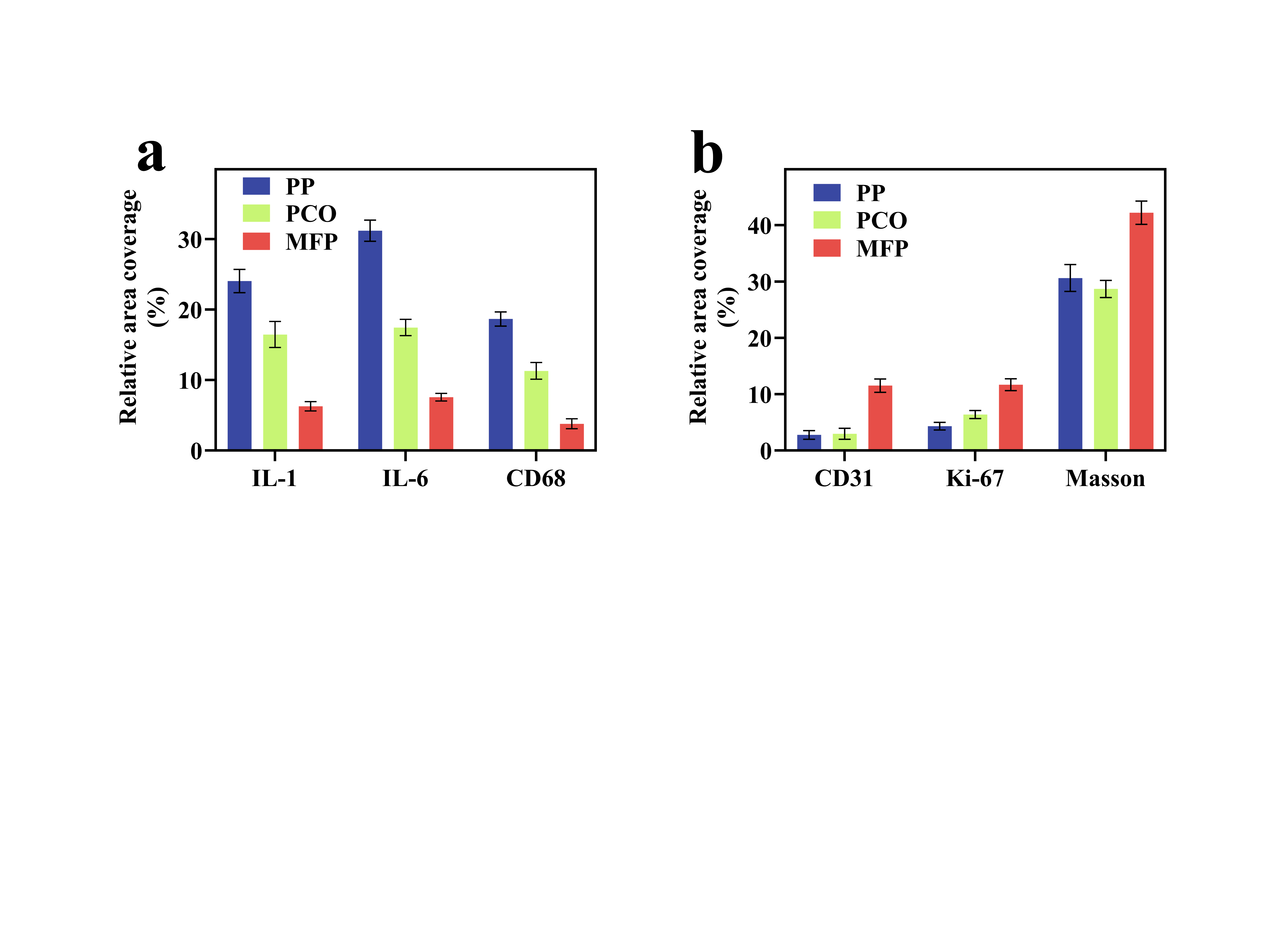

Supplement: Supplementary 1 — Figs. S1 to S19 Table S1 Movies S1 to S4 [file research.0945.f1.zip › Figure S17.TIF]

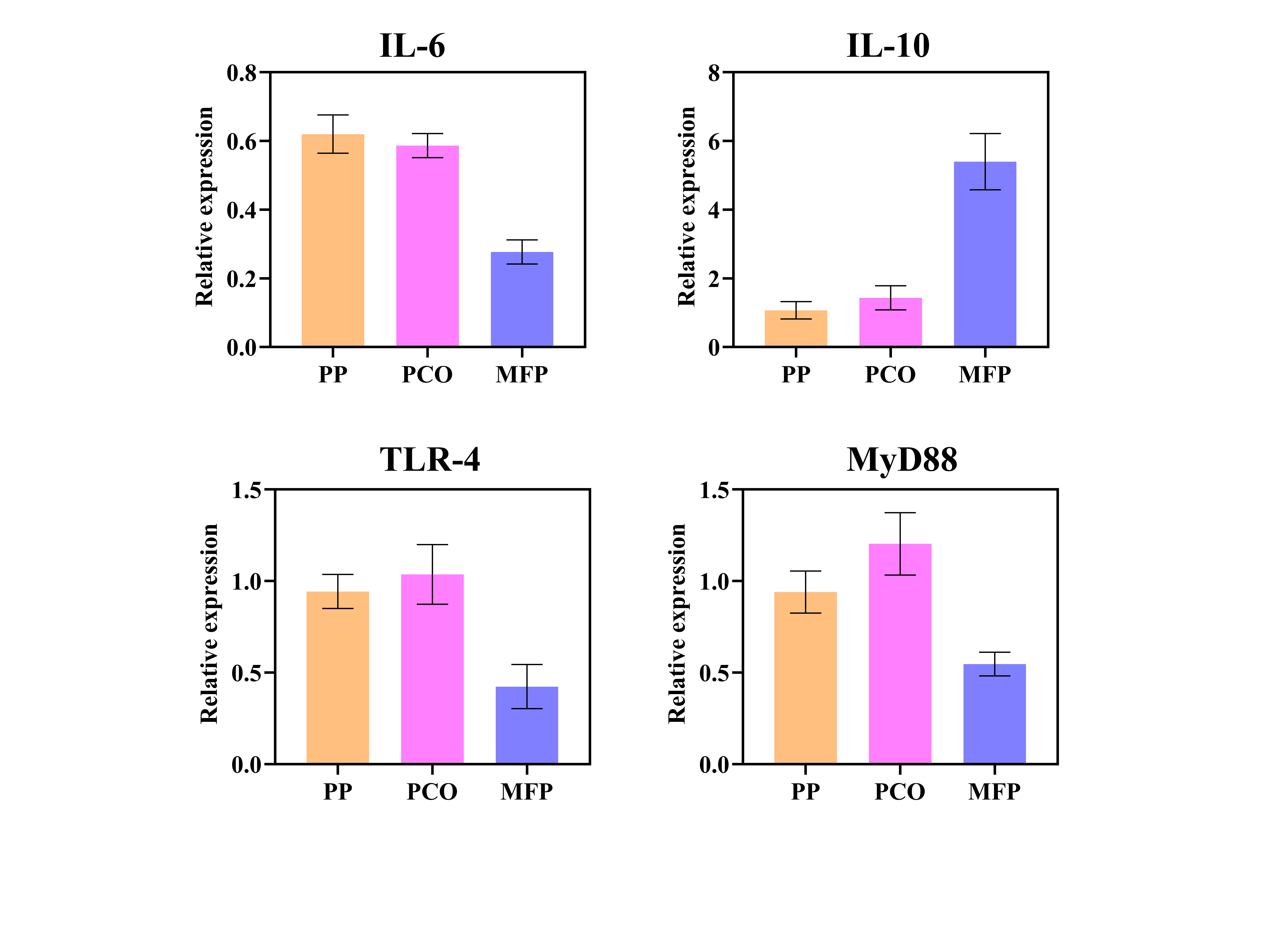

Supplement: Supplementary 1 — Figs. S1 to S19 Table S1 Movies S1 to S4 [file research.0945.f1.zip › Figure S18.TIF]

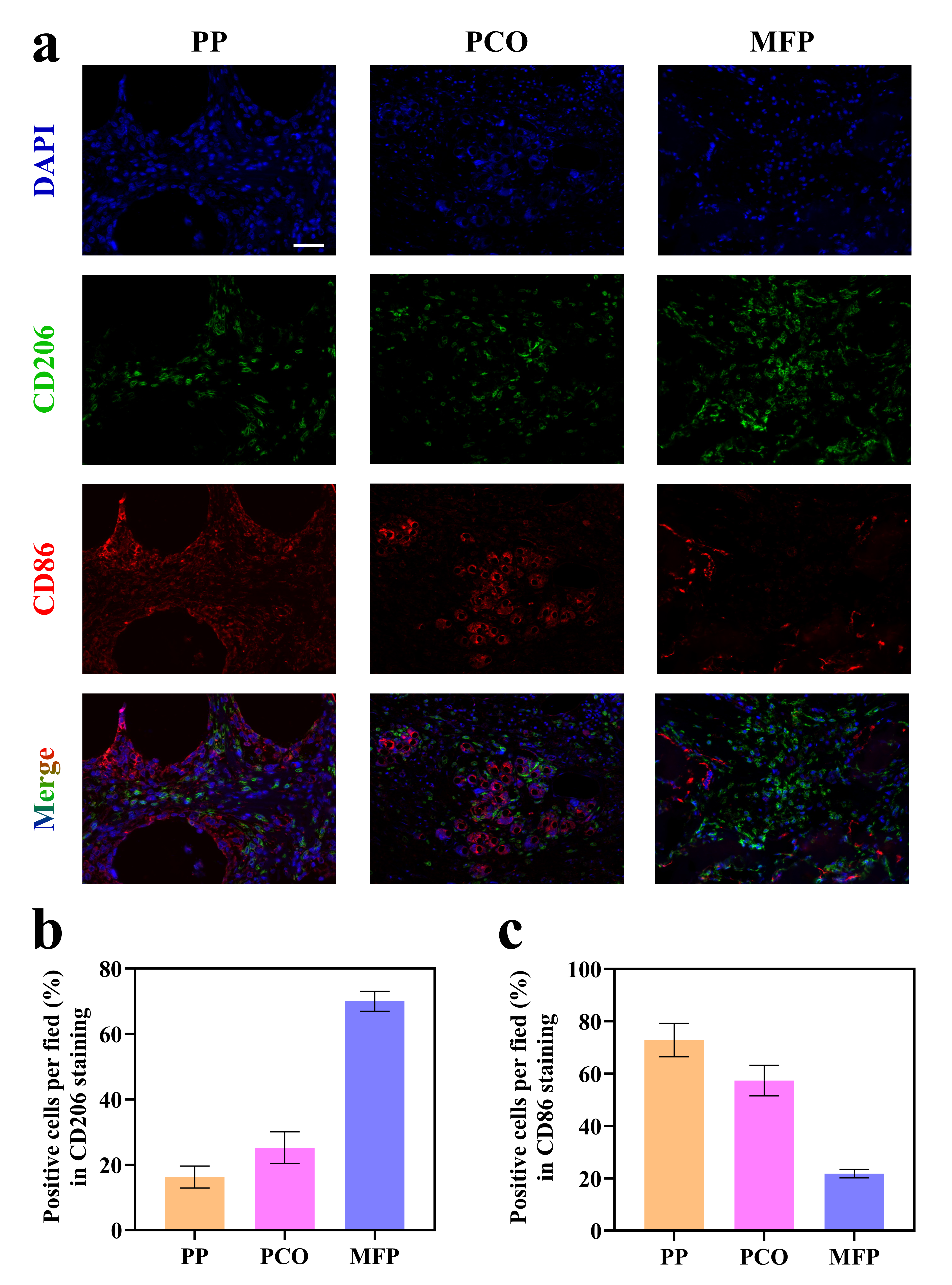

Supplement: Supplementary 1 — Figs. S1 to S19 Table S1 Movies S1 to S4 [file research.0945.f1.zip › Figure S19.tif]

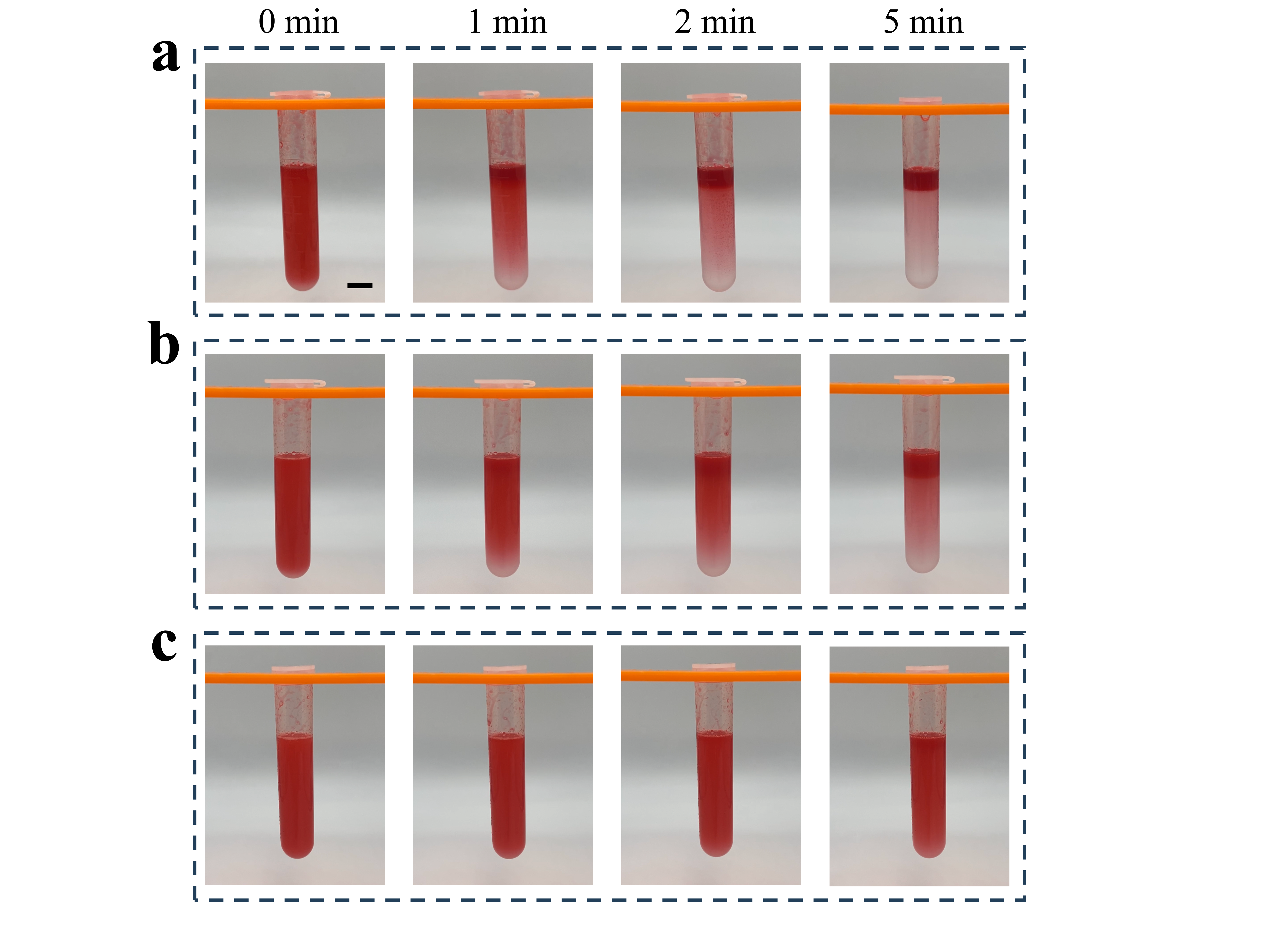

Supplement: Supplementary 1 — Figs. S1 to S19 Table S1 Movies S1 to S4 [file research.0945.f1.zip › Figure S2.TIF]

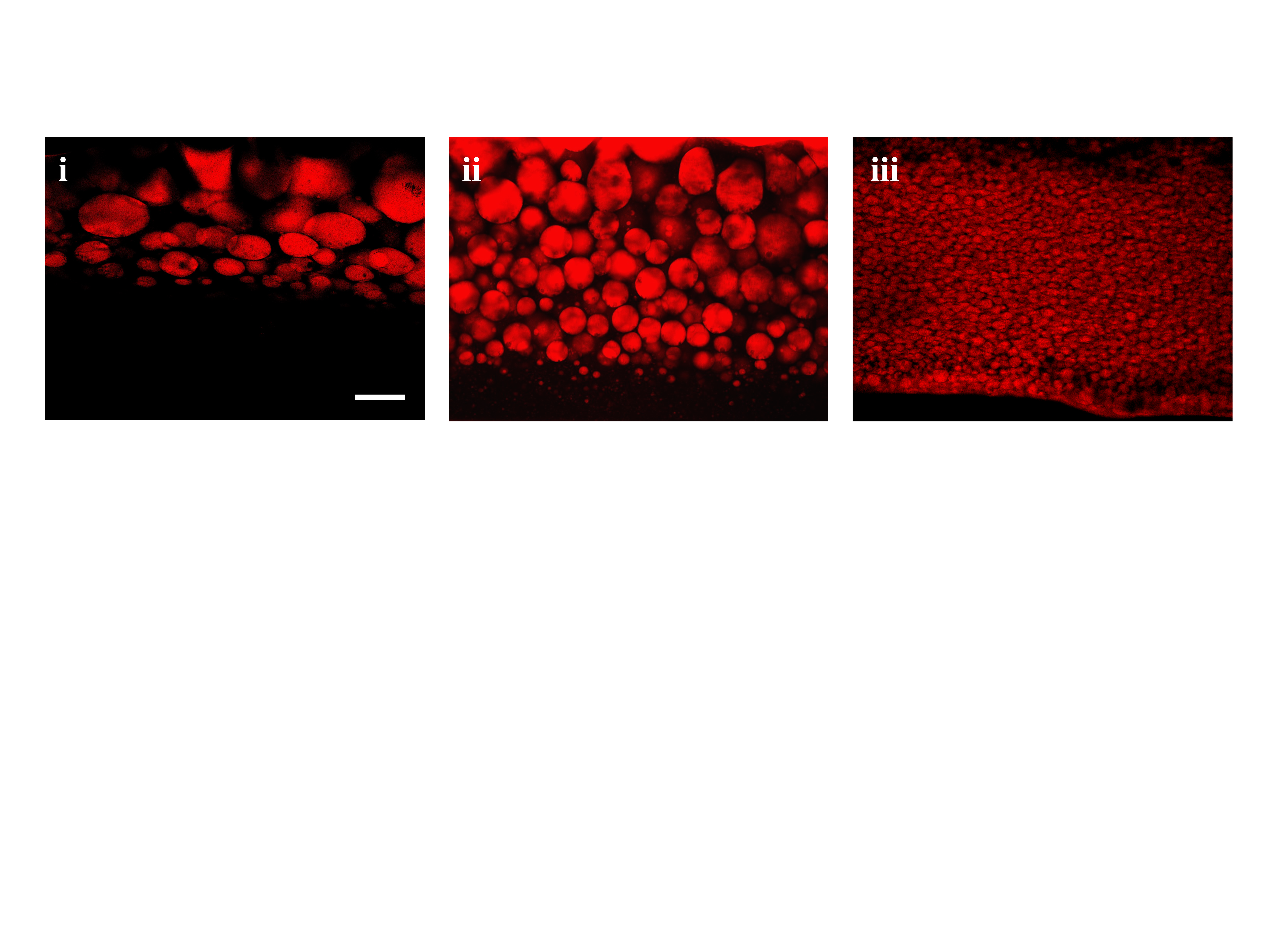

Supplement: Supplementary 1 — Figs. S1 to S19 Table S1 Movies S1 to S4 [file research.0945.f1.zip › Figure S3.TIF]

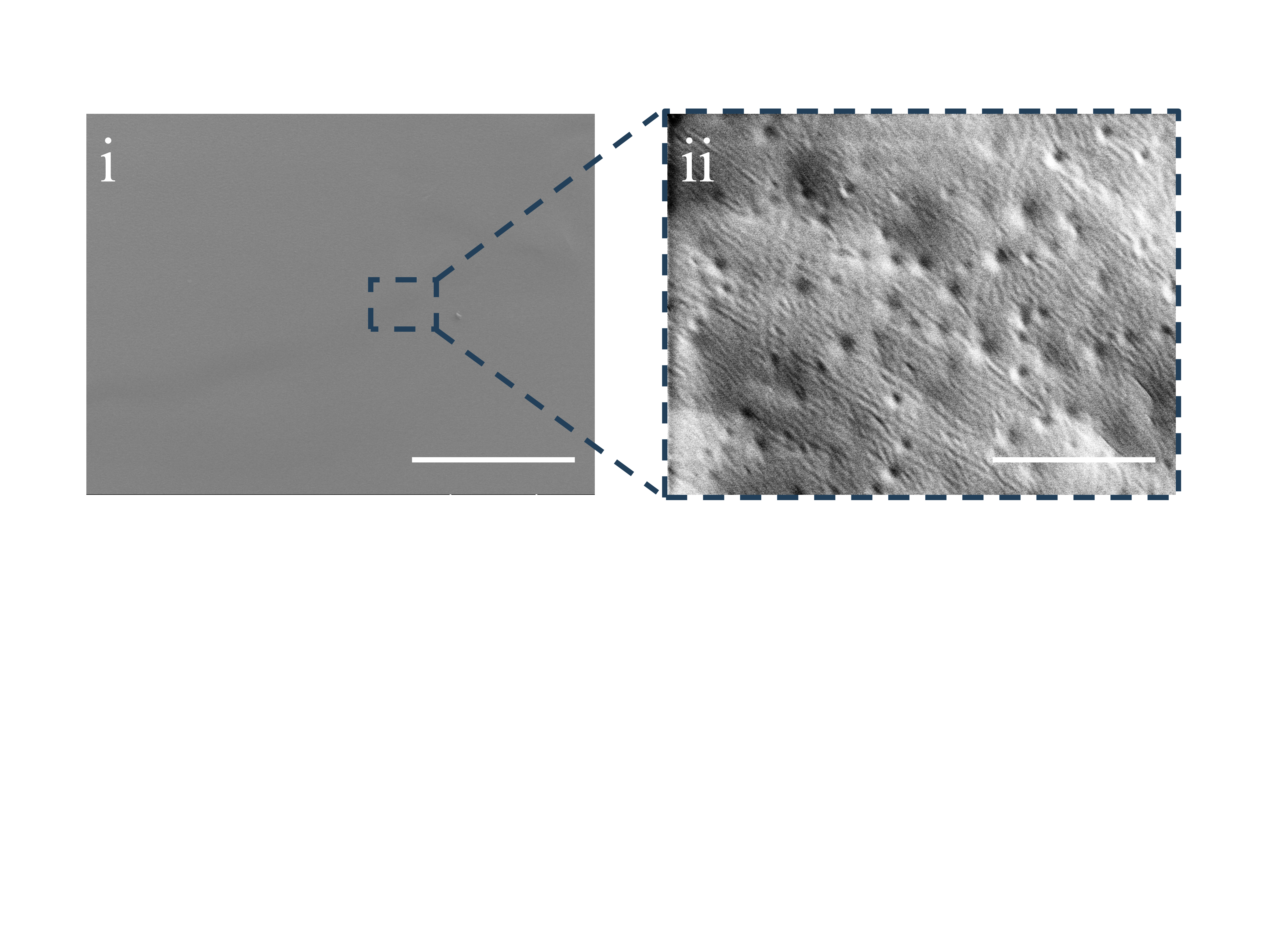

Supplement: Supplementary 1 — Figs. S1 to S19 Table S1 Movies S1 to S4 [file research.0945.f1.zip › Figure S4.TIF]

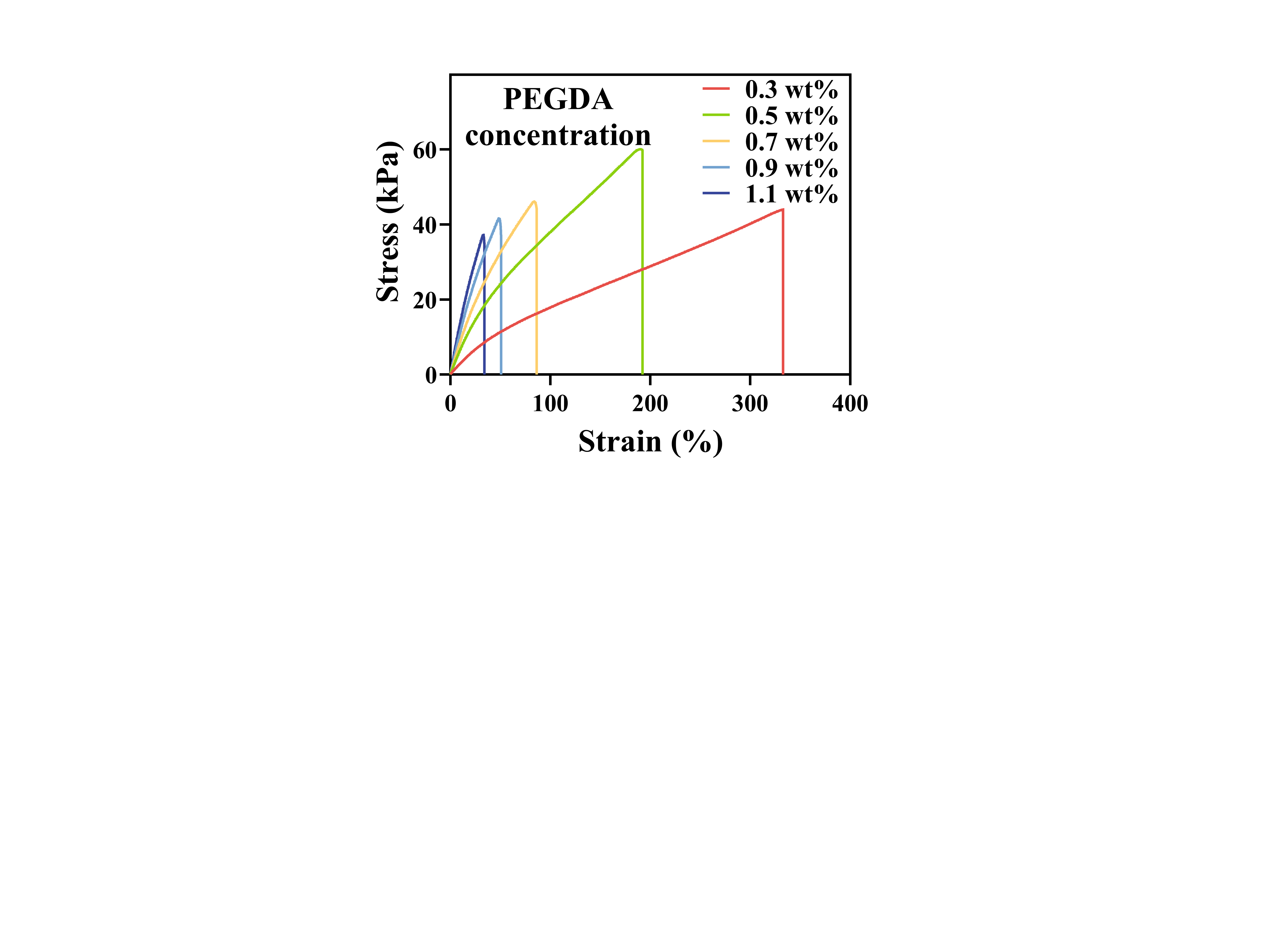

Supplement: Supplementary 1 — Figs. S1 to S19 Table S1 Movies S1 to S4 [file research.0945.f1.zip › Figure S5.TIF]

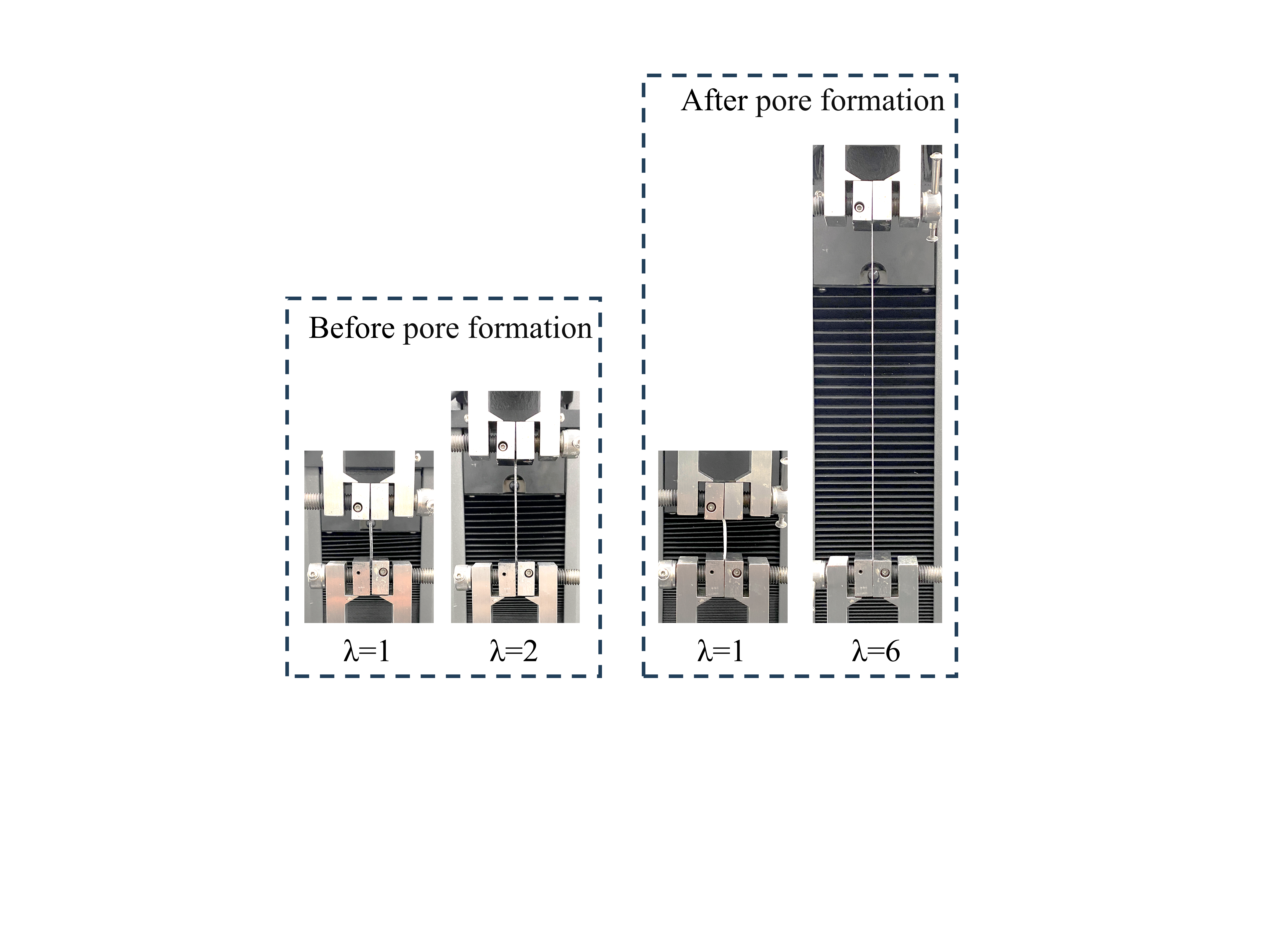

Supplement: Supplementary 1 — Figs. S1 to S19 Table S1 Movies S1 to S4 [file research.0945.f1.zip › Figure S6.TIF]

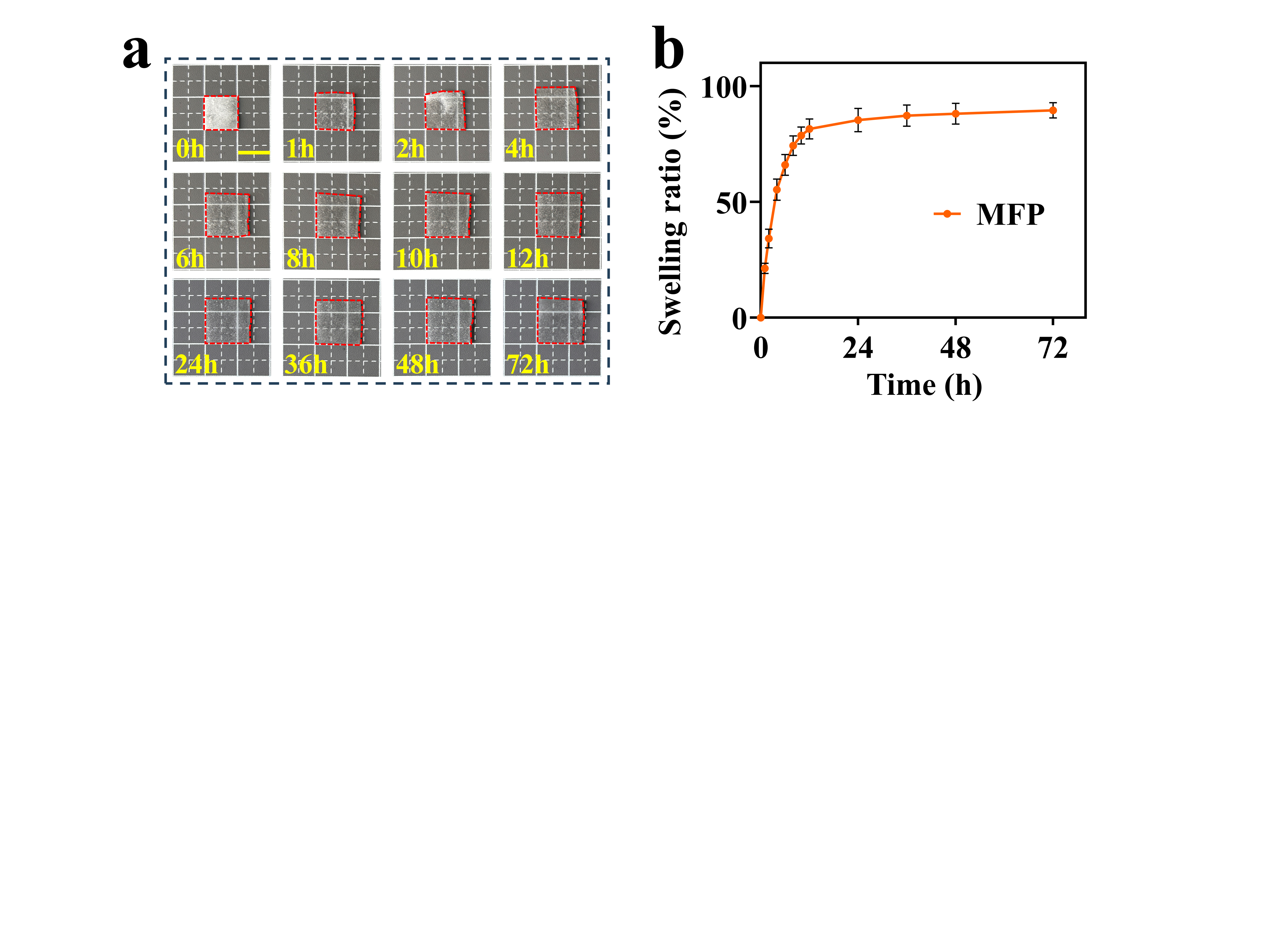

Supplement: Supplementary 1 — Figs. S1 to S19 Table S1 Movies S1 to S4 [file research.0945.f1.zip › Figure S7.TIF]

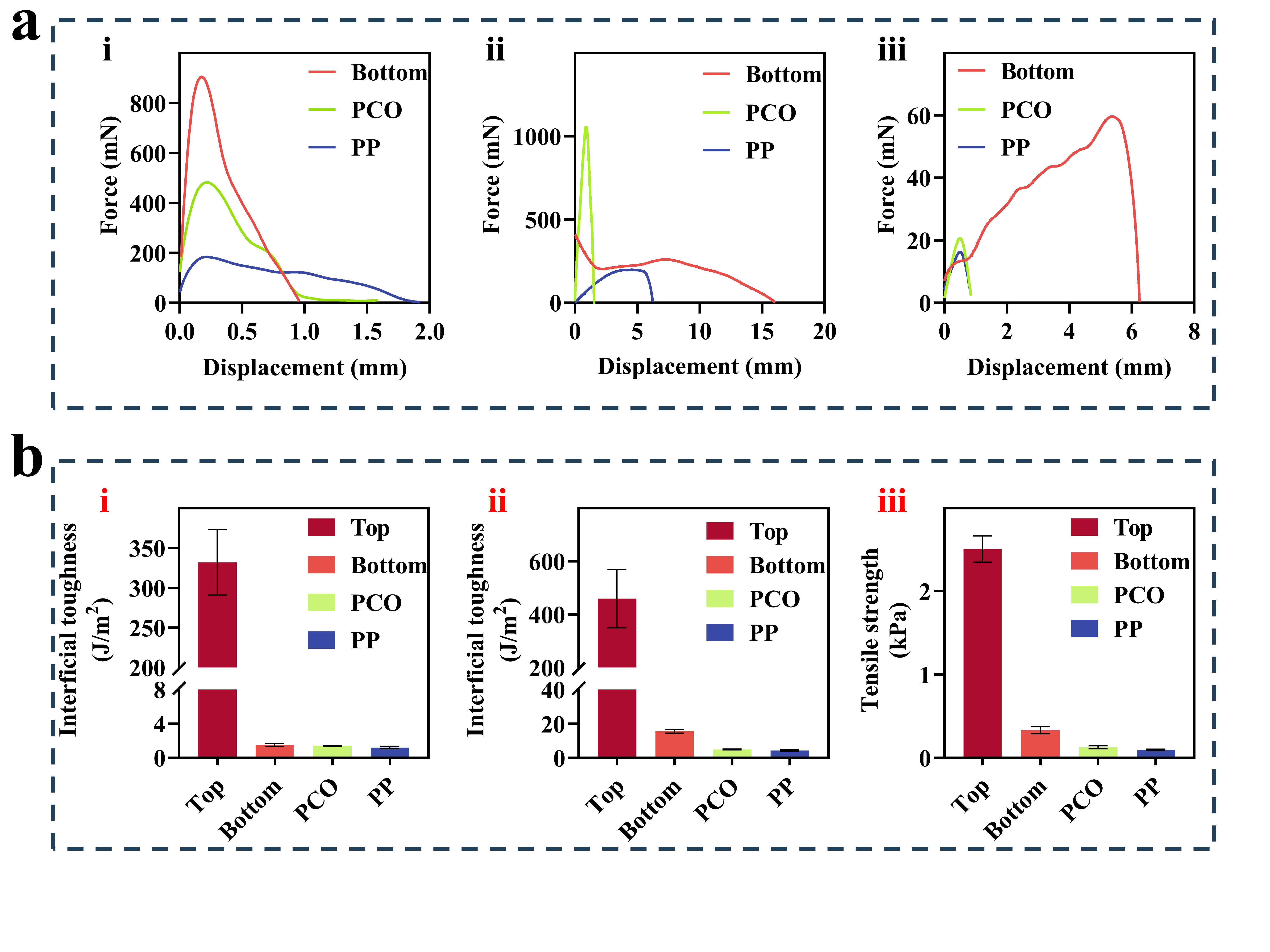

Supplement: Supplementary 1 — Figs. S1 to S19 Table S1 Movies S1 to S4 [file research.0945.f1.zip › Figure S8.TIF]

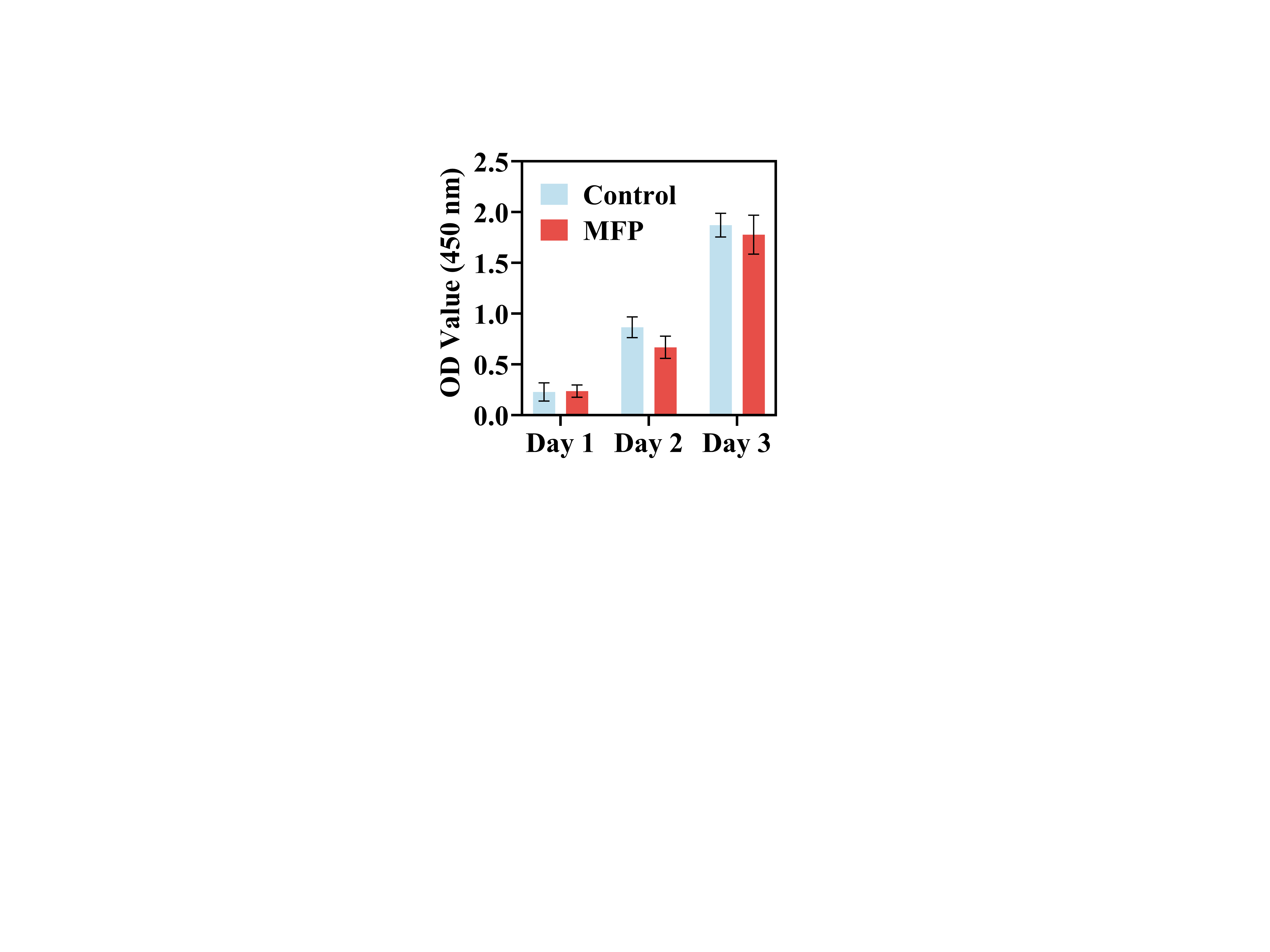

Supplement: Supplementary 1 — Figs. S1 to S19 Table S1 Movies S1 to S4 [file research.0945.f1.zip › Figure S9.TIF]

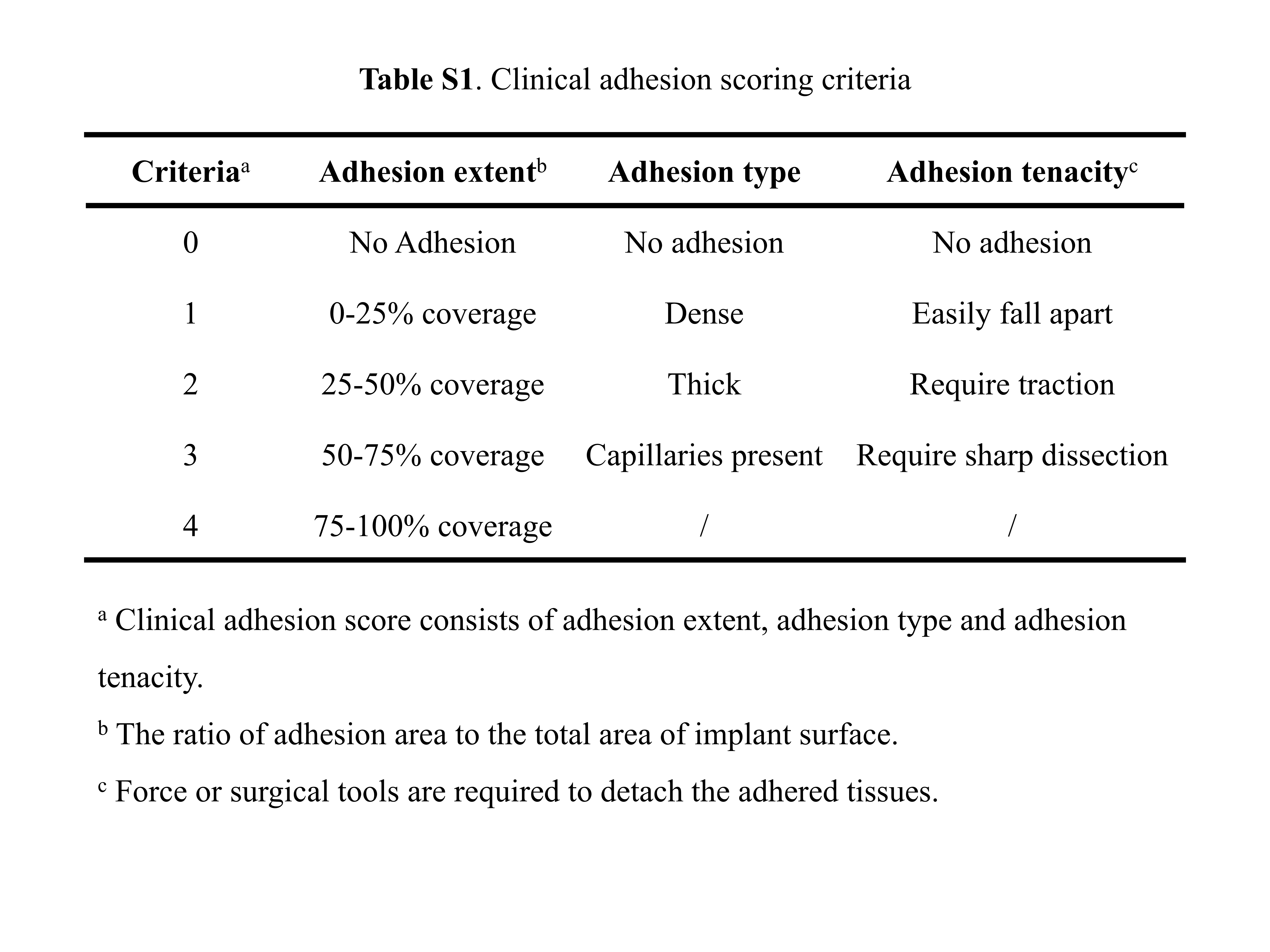

Supplement: Supplementary 1 — Figs. S1 to S19 Table S1 Movies S1 to S4 [file research.0945.f1.zip › Table S1.TIF]
